# Cancer Stemness in *Apc*- vs. *Apc*/*KRAS*-Driven Intestinal Tumorigenesis

**DOI:** 10.1371/journal.pone.0073872

**Published:** 2013-09-17

**Authors:** Mehrnaz Ghazvini, Petra Sonneveld, Andreas Kremer, Patrick Franken, Andrea Sacchetti, Yaser Atlasi, Sabrina Roth, Rosalie Joosten, Ron Smits, Riccardo Fodde

**Affiliations:** 1 Department of Pathology, Josephine Nefkens Institute, Erasmus MC, Rotterdam, The Netherlands; 2 Department of Bioinformatics, Erasmus MC, Rotterdam, The Netherlands; 3 Department of Gastroenterology and Hepatology, Erasmus MC, Rotterdam, The Netherlands; Northwestern University Feinberg School of Medicine, United States of America

## Abstract

Constitutive activation of the Wnt pathway leads to adenoma formation, an obligatory step towards intestinal cancer. In view of the established role of Wnt in regulating stemness, we attempted the isolation of cancer stem cells (CSCs) from *Apc*- and *Apc*/*KRAS*-mutant intestinal tumours. Whereas CSCs are present in *Apc*/*KRAS* tumours, they appear to be very rare (<10^−6^) in the *Apc*–mutant adenomas. In contrast, the Lin^−^CD24^hi^CD29^+^ subpopulation of adenocarcinoma cells appear to be enriched in CSCs with increased levels of active β-catenin. Expression profiling analysis of the CSC-enriched subpopulation confirmed their enhanced Wnt activity and revealed additional differential expression of other signalling pathways, growth factor binding proteins, and extracellular matrix components. As expected, genes characteristic of the Paneth cell lineage (e.g. defensins) are co-expressed together with stem cell genes (e.g. *Lgr5*) within the CSC-enriched subpopulation. This is of interest as it may indicate a cancer stem cell niche role for tumor-derived Paneth-like cells, similar to their role in supporting Lgr5^+^ stem cells in the normal intestinal crypt. Overall, our results indicate that oncogenic *KRAS* activation in *Apc*-driven tumours results in the expansion of the CSCs compartment by increasing **®**-catenin intracellular stabilization.

## Introduction

Colon cancer still represents an ideal model to study the molecular and cellular mechanisms that underlie tumour onset and progression towards malignancy [1], the so-called adenoma-carcinoma sequence [2]. Overall, loss-of-function mutations in the *APC* (adenomatous polyposis coli) tumour suppressor gene or oncogenic mutations in β-catenin (*CTNNB1*) lead to the constitutive activation of Wnt/β-catenin signalling and represents the most common rate limiting (adenoma-forming) events among colon cancer patients. Adenoma growth and progression is often accompanied by alterations in *KRAS* or *BRAF*, followed by loss of the *TP53* and *SMAD4* tumour suppressors thought to underlie the malignant transformation into locally invasive adenocarcinoma [1]. However, this well established genetic evolution model does not take into account other essential characteristics of human colon cancers, namely their cellular heterogeneity (different cell lineages are often present within the primary mass) and the putative role played by a subpopulation of tumour cells, the cancer stem cells (CSCs), in driving tumour growth and determining local invasion into surrounding tissues and distant metastasis [3]. In fact, although the above genetic model would predict that every tumour cell within a colon cancer allegedly initiated by an *APC* or β-catenin mutation should invariably be earmarked by the hallmark of constitutive Wnt activation, namely nuclear β-catenin accumulation, this is only observed in a minority of cells usually located at the invasive front of the primary lesion [4] from where they detach and invade the surrounding stroma [5], [6]. This “β-catenin paradox” nicely illustrates how intra-tumour heterogeneity and possibly tumour stemness ensue at the very initial stages of the adenoma-carcinoma sequence and lead to different Wnt signalling levels among different tumour cells lineages sharing the same (*APC*) mutations [7]. It also indicates that loss of *APC* function (or oncogenic β-catenin activation) is presumably necessary for the onset of the initial dysplastic lesion but insufficient to fully activate Wnt signal transduction and promote malignant transformation in the absence of additional environmental and (epi)genetic factors.

Previously, by employing *in vivo* mutagenesis [8], [9] and gene targeting in the mouse [10], [11], it was shown that loss of *Apc* function results in adenoma formation in the upper GI tract. However, these mouse adenomas fail to progress to malignancy and do not spontaneously accumulate additional genetic hits at the endogenous *Kras* and *Tp53* genes [12]. Notably, whereas oncogenic *KRAS* activation on its own is unable to initiate intestinal tumorigenesis if not with very late onset and only upon somatic hits at the *Tp53* gene [13], compound *Apc* ^1638N/+^/*KRAS* ^V12G^ mice are characterized by a 10-fold increase in tumour multiplicity and by accelerated tumour progression when compared with *Apc* ^1638N/+^ littermates, with the vast majority of the tumour lesion being represented by adenocarcinomas [14]. Further analyses revealed that *Apc* and *KRAS* mutations are synergistic in promoting β-catenin nuclear translocation, thus enhancing canonical Wnt signal transduction [14]. The latter is likely to result from the ability of activated KRAS, through downstream and yet unknown kinases, to induce β-catenin tyrosine phosphorylation thus leading to a substantial increase of its cytoplasmatic pool and its subsequent translocation to the nucleus where it acts as a transcriptional activator of several Wnt downstream target genes. Accordingly, intestinal tumours from *Apc* ^1638N/+^/*KRAS* ^V12G^ mice show a significant increase in cells with nuclear accumulation of β-catenin when compared with *Apc* ^1638N/+^ animals [14].

In recent years, CSCs have been successfully purified from human colon cancers by employing different cell surface markers such as CD133 [15], [16], EpCAM, CD44 and CD166 [17], and EphB2 [18]. However, although the above cell surface antigens have been instrumental for the identification of tumour cell subpopulations with enriched tumour-initiating properties when transplanted into immune-incompetent recipient mice, our understanding of the mechanisms underlying intestinal cancer stemness and of the role played by CSCs in progression towards malignancy is still largely incomplete. Previously, Vermeulen et al. showed that high Wnt activity earmarks CSCs within suspension spheres derived from colon tumors [19]. From this perspective, a number of additional issues need to be addressed: is nuclear β-catenin accumulation a functional marker for intestinal CSCs *in vivo*? Are tumor cells with stem-like properties already present in early, benign lesions such as adenomas? Here, we took advantage of the *Apc* ^1638N/+^ and *Apc* ^1638N/+^/*KRAS* ^V12G^ mouse models for intestinal tumorigenesis to prospectively identify subpopulations of tumour stem-like cells and characterize them with regard to their multipotency, self-renewal, genome-wide expression profile, and Wnt/β-catenin signalling activity.

## Results

### Tumour-initiating Cells are Present in *Apc^1638N/+^/KRAS^V12G^* but are Very Rare in *Apc^1638N/+^* Intestinal Tumours

The *Apc* ^1638N/+^ mouse model develops an average of 4–5 benign upper GI tumours (adenomas) in the C57Bl/6J genetic background [10], [11]. These lesions only rarely (and with late onset) develop into adenocarcinomas as also shown by the lack of spontaneous somatic mutations occurring at the *Kras* and *Tp53* genes [12]. To assess the presence of tumour-initiating cells in *Apc* ^1638N/+^ adenomas, i.e. cells capable of recapitulating the primary lesion when transplanted into a recipient animal, intestinal tumours were collected from *Apc* ^1638N/+^ animals and dissociated both mechanically and enzymatically into single cell suspensions and depleted from endothelial and hematopoietic cells (Lin^+^) by fluorescence-activated cell sorting (FACS). Next, different multiplicities of the resulting Lin^−^ population of bulk tumour cells were transplanted subcutaneously into NOD-SCID animals. As shown in Table 1, no tumour growth was observed even upon injection of as many as 0.5–1.0*10^6^ Lin^-^ cells and 6 months after transplantation.

**Table 1:** Subcutaneous transplantation of bulk intestinal tumor cells in immune-incompetent mice indicates the presence of CSCs in *Apc* ^1638N/+^/*KRAS* ^V12G^ but not in *Apc* ^1638N/+^ tumours.

*Sorted tumor* *population*	*No. of* *transplanted cells*	*Tumors in* *NOD/SCID*
*Apc* ^1638N/+^ Lin^−^ (bulk)	1.0×10^6^	0/1
*Apc* ^1638N/+^ Lin^−^ (bulk)	5.0×10^5^	0/7
*Apc* ^1638N/+^ Lin^−^ (bulk)	3.0× 10^5^	0/6
*Apc* ^1638N/+^ Lin^−^ (bulk)	2.5×10^5^	0/6
*Apc* ^1638N/+^ Lin^−^ (bulk)	1.0×10^5^	0/12
*Apc* ^1638N/+^/*KRAS* ^V12G^Lin^−^ (bulk)	1.0×10^5^	23/33
*Apc* ^1638N/+^/*KRAS* ^V12G^Lin^−^ (bulk)	5.0×10^3^	2/16
*Apc* ^1638N/+^/*KRAS* ^V12G^Lin^−^ (bulk)	1.5×10^3^	3/48

Next, we repeated the transplantation assay with bulk Lin^−^ tumour cells from *Apc* ^1638N/+^/*KRAS* ^V12G^ intestinal tumours. As previously reported, the majority of the intestinal tumours found in these compound animals in the same inbred C57B6/J genetic background are locally invasive adenocarcinomas [14]. In sharp contrast with the Lin^-^ cells from *Apc* ^1638N/+^ adenomas, tumour growth was observed in 23 out of 33 injections with 10^5^ *Apc* ^1638N/+^/*KRAS* ^V12G^ Lin^-^ cells and, though at lower incidence (3 out of 48 transplantations), even with as low as 1500 cells (Table 1). By limiting dilution analysis (L-Calc™) the frequency of tumour-initiating cells in the Lin^−^ population from *Apc* ^1638N/+^/*KRAS* ^V12G^ intestinal tumours was estimated as 1 in 72838 (95% CI of 109467 to 48465). As for the *Apc* ^1638N/+^ adenomas, tumour-initiating cells are likely to be present, if at all, at considerably lower frequencies (<10^−6^). Hence, the presence of tumour-initiating cells, as defined by transplantation assays, appears to be limited to the tumours from *Apc* ^1638N/+^/*KRAS* ^V12G^ mice when compared with those from the *Apc* ^1638N/+^ mouse model.

### The Lin-CD24^hi^CD29^+^ Subpopulation from *Apc^1638N/+^/KRAS^V12G^* Intestinal Tumours Encompass Tumour-initiating and Self-renewing CSCs

**Figure 1:**
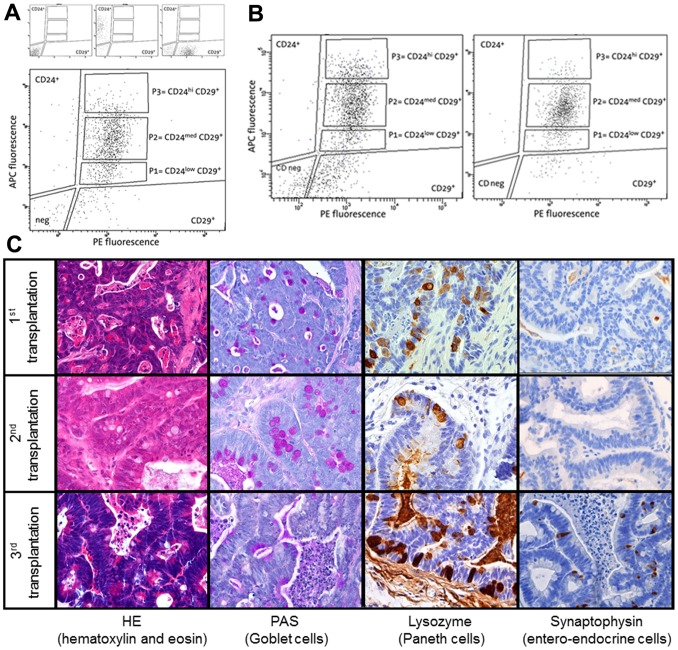
FACS analysis of cell suspensions from *Apc* ^1638N/+^/*KRAS* ^V12G^ tumours. **a.** Large panel: dot plot representative of the staining pattern obtained by staining with anti-CD24 APC-conjugated and anti-CD29 PE-conjugated antibodies. Lineage positive cells (Lin^+^) were excluded (gated out) by staining with biotinylated antibodies against lineage markers and Streptavidin-PerCPCy5.5. P1 (Lin^−^CD24^low^CD29^+^), P2 (Lin^−^CD24^med^CD29^+^), and P3 (Lin^−^CD24^hi^CD29^+^) populations are indicated in the plot. Small panels: dot plots representative of cells stained with isotypic control antibodies (left), compensation control stained only with anti CD24-APC antibodies (middle), compensation control stained only with anti CD29-PE antibodies (right). **b.** FACS analysis of the CD24/CD29 pattern of tumours obtained by serial transplantation of P3 cells suspensions from *Apc* ^1638N/+^/*KRAS* ^V12G^ intestinal tumours. Left: primary transplantation. Right: secondary transplantation. **c.** Immunohistochemistry analysis of tumors obtained by 3 rounds of serial transplantation of P3 cells suspensions from *Apc* ^1638N/+^/*KRAS* ^V12G^ intestinal tumours.

In order to prospectively enrich and eventually isolate tumour-initiating cells from the bulk Lin^-^ population of *Apc* ^1638N/+^/*KRAS* ^V12G^ tumours, we first tested a panel of previously established (cancer) stem cell markers including CD24, CD29 (β1 integrin), CD44, CD97, and L1CAM by FACSorting and subsequent transplantation in NOD-SCID mice. In contrast to CD44, L1CAM, and CD97 (Table S1), transplantation of Lin^-^CD24^+^CD29^+^ cells revealed a slight yet significant enrichment in tumour-initiating cells (estimated frequency of 1 in 56463, with CI of 399211 to 7986) (Table 2). Given that the Lin^-^CD24^+^CD29^+^ population represents a relatively large proportion of the bulk cells (∼80%; data not shown), we then further defined three additional FACS gates based on the relative expression of the CD24 cell surface antigen (CD24^hi^, CD24^med^, and CD24^low^) (Figure 1a). Out of 30 primary *Apc* ^1638N/+^/*KRAS* ^V12G^ tumours analysed by FACS, the average size (expressed in percentage of the bulk Lin^−^ fraction) of each CD24/CD29 sorted subpopulation was determined: CD24^−^CD29^−^, 3.4% (SD 2.6); CD24^−^CD29^+^, 7.8% (SD 4.7); CD24^+^CD29^−^, 4.4% (SD 3.4); CD24^lo^CD29^+^ (P1), 7.9% (SD 2.5); CD24^med^CD29^+^ (P2), 52.9% (SD 7.4); CD24^hi^CD29^+^ (P3), 10.0% (SD 4.7). Please note that these percentages do not add to 100% simply because of the deliberate exclusion of cells located in the separations between sorting gates (see Figure 1a).

**Table 2:** Subcutaneous transplantation of sorted cells from *Apc* ^1638N/+^/*KRAS* ^V12G^ tumours in immune-incompetent mice indicates the presence of tumor-initiating cells in the CD24^hi^CD29^+^ subpopulation.

*Sorted tumor* *population*	*No. of* *transplanted cells*	*Tumors in* *NODSCID*
Lin^−^CD24^+^CD29^+^	5.0×10^3^	8/24
Lin^−^ depletedof CD24^+^CD29^+^	5.0×10^3^	1/10
Lin^−^CD24+CD29^+^	1.5×10^3^	3/30
Lin^−^ depletedof CD24^+^CD29^+^	1.5×10^3^	0/6
Lin^−^CD24^low^CD29^+^	1.5×10^3^	1/28
Lin^−^CD24^med^CD29^+^	1.5×10^3^	0/28
Lin^−^CD24^hi^CD29^+^	1.5×10^3^	13/28

The frequency of tumour-initiating cells within the P1, P2 and P3 subpopulations was determined by transplanting 1500 cells of each into NOD-SCID mice. Notably, whereas at this multiplicity CD24^med^CD29^+^ and CD24^lo^CD29^+^ cells consistently failed to form tumours (only 1 growth out of 56 transplantations), the Lin^-^CD24^hi^CD29^+^ subpopulation was found to encompass a substantial enrichment in tumorigenic cells (13/28; Table 2). Hence, although in this case we could not perform limiting dilution analysis by L-Calc™ (since a fixed multiplicity of cells was employed) the Lin^-^CD24^hi^CD29^+^ tumour subpopulation appears to be characterized by a significant relative enrichment of approx. 20–25 fold when compared with total Lin^-^ bulk cells (1 CSC out of ∼3000 tumour cells vs. 1 in 72838).

The definition of cancer stem cells cannot be exclusively based on their ability to form tumours when transplanted at low multiplicity in immune-incompetent mice. Equally important characteristics of CSCs are their unique ability to self-renew and differentiate to fuel and recapitulate the heterogeneous composition of the primary tumour they are derived from. In order to determine whether the tumour-initiating cells encompassed by the Lin^-^CD24^hi^CD29^+^ population are also capable of self-renewal and differentiation, we performed serial transplantations experiments. First, 1500 Lin^-^CD24^hi^CD29^+^ cells were isolated from *Apc* ^1638N/+^/*KRAS* ^V12G^ primary intestinal tumours and transplanted in NOD-SCID recipient mice. As anticipated from our previous results, this initial assay gave rise to subcutaneous tumours within 8 to 10 weeks. The resulting tumours were then excised from the recipient NOD-SCID animals and employed for both FACS and histological analysis. As for FACS, tumours were dissociated into single cell suspensions and analysed, sorted and transplanted according to their CD24 and CD29 expression levels. Secondary tumours originated from Lin^-^CD24^hi^CD29^+^ cells fully recapitulated the CD24/CD29 FACS expression profile of the primary lesions (Figure 1b and Figure S2). Likewise, upon transplantation of 1500 cells from each of the Lin^−^CD24CD29 populations obtained from the secondary tumours, only the Lin^−^CD24^hi^CD29^+^ cells were capable of forming tertiary tumours. Accordingly, the FACS profile of the tertiary tumours recapitulates that of the primary lesions (Figure 1b). Notably, immunohistochemistry (IHC) and enzymatic staining revealed a progressive increase in the relative presence of intestinal differentiation lineages, namely Goblet (Periodic Acid Schiff; PAS), Paneth (lysozyme), and entero-endocrine (synaptophysin) cells in the transplanted intestinal tumours when compared with the primary *Apc* ^1638N/+^/*KRAS* ^V12G^ lesions (Figure 1c). However, FACS analysis of the serially transplanted tumours showed that the relative size of the individual CD24/CD29 subpopulations did not significantly changed (Figure S3).

Thus, the Lin^-^CD24^hi^CD29^+^ subpopulation of tumour cells from *Apc* ^1638N/+^/*KRAS* ^V12G^ adenocarcinomas encompass *bona fide* CSCs with tumour-initiating, self-renewing and differentiation capacities.

### Lin-CD24^hi^CD29^+^ Cells from *Apc^1638N/+^/KRAS^V12G^* Intestinal Tumours Show Increased Intracellular β-catenin Accumulation

**Figure 2:**
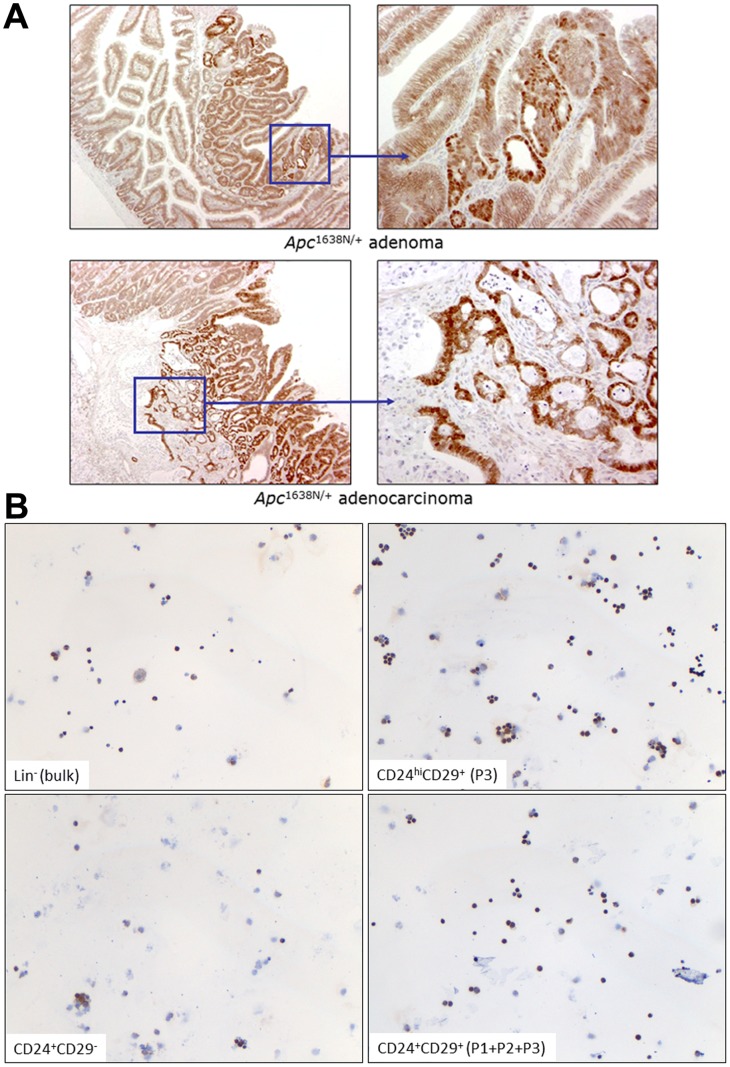
β-catenin expression analysis in *Apc* ^1638N/+^ and *Apc* ^1638N/+^/*KRAS* ^V12G^ intestinal tumours. Immuno-histochemistry (**a.**,**b.**) and western blot (**c.**) analysis of β-catenin in primary *Apc* ^1638N/+^ intestinal adenomas (**a.**) and in FACSorted tumour populations from *Apc* ^1638N/+^/*KRAS* ^V12G^ intestinal tumours (**b.** and **c.**). The bars in **c.** represents the quantification of the bands obtained with an anti-active β-catenin Ab (anti-ABC; clone 8E7, #05–665, Millipore) by scanning and analyzing the western blot with the Odyssey scanner and after normalization with β-actin.

We previously proposed that the minority of colon cancer cells featuring nuclear β-catenin accumulation and non-randomly distributed along the invasive front, represent CSCs [7]. Notably, both *Apc* ^1638N/+^ adenomas and *Apc* ^1638N/+^/*KRAS* ^V12G^ carcinomas share the same “β-catenin paradox” observed in human colon cancers in that, upon IHC analysis, only a minority of tumour cells show nuclear β-catenin accumulation notwithstanding that the majority, if not all, share the two-hits at the *Apc* locus [12], [14] (Figure 2a). To assess whether the CSCs enriched in the Lin^−^CD24^hi^CD29^+^ tumour subpopulation are characterized by an increased level of intracellular β-catenin, we analysed protein expression in the different FACSorted tumour cell subpopulations by two independent assays, namely immuno-staining and western blot analysis. Immuno-staining showed that the majority of Lin^-^CD24^hi^CD29^+^ intestinal tumour cells are characterized by intracellular accumulation of β-catenin when compared with other sorted populations and the bulk (Lin^−^) tumour cells (Figure 2b). This result was also confirmed in a more quantitative fashion by western analysis performed with antibodies specific for the signalling-competent fraction (i.e. dephosphorylated at residues Ser37 and Thr41) of the β-catenin protein (Figure 2c and Figure S4).

Overall, these data confirm that the Lin^-^CD24^hi^CD29^+^ subpopulation from *Apc* ^1638N/+^/*KRAS* ^V12G^ tumours, here shown to be enriched in CSCs, encompasses a significantly higher level of intracellular and signalling-competent β-catenin when compared to bulk Lin^−^ and other tumour cell subpopulations. The increased Wnt signalling activity in CSCs was later confirmed by expression profiling and Taqman qPCR analysis of Wnt downstream target genes (e.g. *Lgr5*, *Axin2*, *T*, and *Lef1*; see here below).

### The Expression Signature of CSCs from *Apc^1638N/+^/KRA^SV12G^* Intestinal Tumours is Distinct from that of Differentiated and Bulk Tumour Cells and Encompasses Both Stem and Paneth Cell Markers

To identify molecular differences between stem-like and more differentiated (bulk) tumour cells from *Apc* ^1638N/+^ and *Apc* ^1638N/+^/*KRAS* ^V12G^ intestinal tumours, we isolated total RNA from 10^4^ Lin^-^CD24^hi^CD29^+^ (P3), Lin^-^CD24^med^CD29^+^/Lin^−^CD24^lo^CD29^+^ (P1+P2, merged gate) and Lin^-^ (bulk) tumour cells from 5 individual mice of each genotype (*Apc* ^1638N/+^ and *Apc* ^1638N/+^/*KRAS* ^V12G^). Total RNA samples were then employed to hybridize oligonucleotide microarrays (Affymetrix Mouse Genome 430A 2.0 Array) according to conventional protocols.

First, the different tumour cell populations isolated from mice with different genotypes (*Apc* ^1638N/+^ and *Apc* ^1638N/+^/*KRAS* ^V12G^) were compared by ANOVA (3 ways) with a FDR (false discovery rate) set at 0.05 to select for genes with ≥2-fold differential expression. Notably, the P3 (CSCs from both genotypes) vs. Lin^−^ (bulk; both genotypes) and P3 vs. P1+P2 comparisons resulted in significant differences and in the definition of two lists of differentially expressed probe sets (n = 1062 [851 non-redundant, annotated genes] and 746 [602 non-redundant, annotated genes], respectively; Tables S3 and S4). In this fashion, we identified a list of 587 differentially expressed probe sets from the intersection of the Lin- vs. P3 and P1+P2 vs. P3. The identified probe sets correspond to 482 genes (non-redundant) (Table S5).

**Figure 3:**
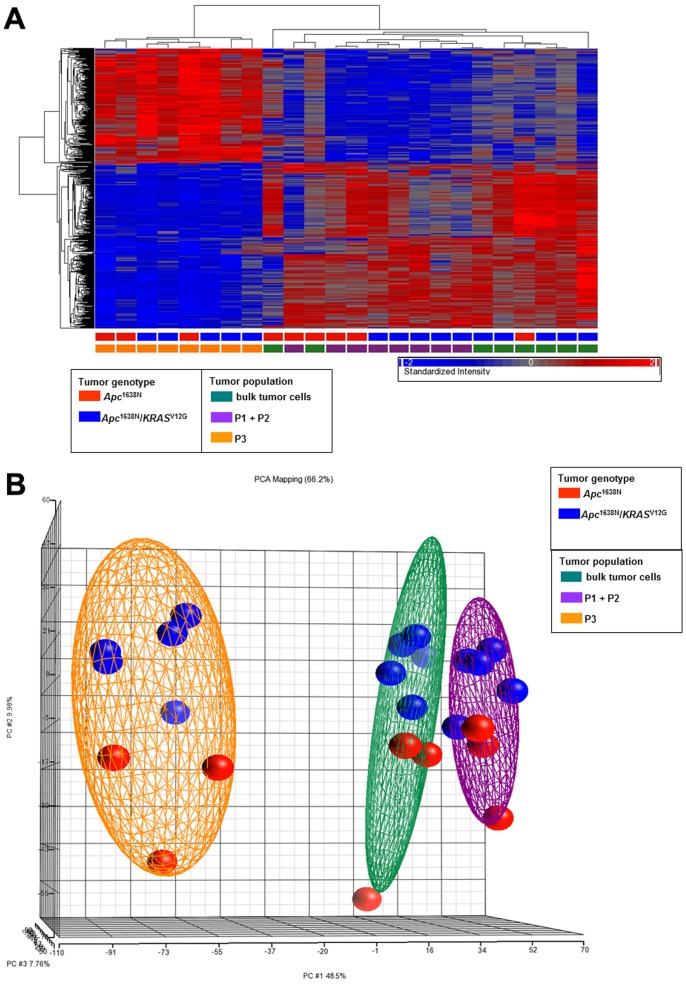
Expression profiling analysis of tumor cell subpopulations from *Apc* ^1638N/+^ and *Apc* ^1638N/+^/*KRAS* ^V12G^ intestinal tumours. (a.) Hierarchical clustering and (b.) Principal Components Analysis (PCA) (both implemented in Partek) of Lin^−^CD24^hi^CD29^+^ (P3), Lin^−^CD24^med^CD29^+^/Lin^−^CD24^lo^CD29^+^ (P1+P2, merged gate) and Lin^−^ (bulk) tumor cells from 5 individual mice of each genotype (*Apc* ^1638N/+^ and *Apc* ^1638N/+^/*KRAS* ^V12G^). For better visualization individual colours were used for each group and in b. ellipsoids were drawn around the three tumour populations.

The expression profiles obtained from the CSC-enriched subpopulations (Lin^−^CD24^hi^CD29^+^) of both genotypes (*Apc* ^1638N/+^ and *Apc* ^1638N/+^/*KRAS* ^V12G^) differ from those of more differentiated (Lin^−^CD24^med^CD29^+^/CD24^lo^CD29^+^) and of the Lin^−^ bulk subpopulations as clearly visible in the hierarchical clustering (HCA) and principal component (PCA) analysis (Figure 3).

Notably, the *Apc*- and *Apc*/*KRAS*-mutant genotypes are not resolved by HCA and PCA, possibly due to the relatively limited number of *Apc* ^1638N/+^ and *Apc* ^1638N/+^/*KRAS* ^V12G^ tumour samples (n = 5 for each group) employed for the comparative expression profiling analysis, insufficient to highlight the allegedly more subtle differences between benign and malignant CSCs.

Next, from the above lists of differentially expressed probes between the P3 and other tumour cell populations we validated the expression of a total of 35 genes by quantitative real-time PCR (Table S2). The selection of validated genes includes, apart from the top up- and down-regulated genes, also additional members of the canonical Wnt signal transduction pathway, and genes known to play relevant roles in cancer. Overall, the vast majority of the selected genes (33/35) were validated for their differential expression by qPCR (Figure S1).

Gene ontology analysis [20] of the intersection signature revealed a rather broad spectrum of cellular functions, structures and processes among the up-regulated genes including extracellular matrix, cell adhesion, organ development and morphogenesis (Table S6). In particular, analysis of the genes differentially expressed in the CD24^hi^CD29^+^ cells from *Apc* ^1638N/+^ and *Apc* ^1638N/+^/*KRAS* ^V12G^ tumours in comparison with bulk and more differentiated tumour cells, revealed several biological processes likely to play functional roles in cancer stemness. First, as also shown by the intracellular accumulation of active β-catenin, several targets and members of the Wnt signalling cascade are differentially expressed in the P3 signature including *Lgr5*, *Mmp2* and *Mmp7*, *Dkk2*, *Pla2g2a*, *Prox1*, *Sox17*, *T* (brachyury), *Wif1*, and *Fzd5*. The presence of *Lgr5* among the upregulated genes is of interest as it indicates that this well-known marker of normal cycling stem cells in the mouse intestine [21] might also represent a useful CSC marker in mouse intestinal tumours as recently demonstrated by lineage tracing [22]. Also, the transcription factor *Prox1*, a direct and dose-dependent target of the Wnt/β-catenin signalling pathway, was upregulated in the P3 population. *Prox1* was previously shown to promote dysplasia in colonic adenomas and colorectal cancer progression [23]. However, we could not find any significant differences between *Prox1* expression levels between *Apc* ^1638N/+^ and *Apc* ^1638N/+^/*KRAS* ^V12G^ tumour cells both in the original microarray data and upon qPCR validation (Figure S1). This observation reflects a more general lack of significant differences between the P3 populations of *Apc* ^1638N/+^ and *Apc* ^1638N/+^/*KRAS* ^V12G^ tumours, as also shown by hierarchical clustering and PCA analysis (Figure 3a and b, respectively). In our previous study [14], expression profiling of bulk tumours from *Apc* ^1638N/+^ and *Apc* ^1638N/+^/*KRAS* ^V12G^ mice also did not resolve the two genotypes. In fact, only the relative number of tumour cells with nuclear β-catenin could significantly distinguish between *Apc* ^1638N/+^ from *Apc* ^1638N/+^/*KRAS* ^V12G^ intestinal tumours [14].

Apart from Wnt, additional signalling pathways are represented by the differentially regulated genes as shown by the differential expression of *Bmp7* and *Bmper* (Bmp signaling), *Fgfbp1*, *Fgfrl1*, and *Etv5* (fibroblast growth factor receptors, binding proteins and transcription factors), and *Igf1*, *Igfbp1*, *Igfbp5*, and *Igfbp7* (insulin-like growth factors and binding proteins).

Overall, these results show that CSCs from *Apc*- and *Apc*/*KRAS*-mutant tumours have distinct expression profiles from other tumour cell populations and are characterized by increased Wnt signalling activity, in agreement with their enhanced levels of intracellular β-catenin, together with other signalling pathays (Bmp, Igf), and by the expression of Paneth cell-specific genes.

## Discussion

Mutations in the *APC* tumour suppressor gene represent the main initiating and rate-limiting event in the adenoma-carcinoma sequence leading to colon cancer in man [1]. Loss of *APC* function leads to the constitutive activation of the canonical Wnt/β-catenin signalling pathway known to play crucial roles in the regulation of self-renewal and differentiation in a broad spectrum of tissue-specific stem cell niches including the intestinal crypt and, accordingly, in the onset of many cancer types [24]. Constitutive Wnt signalling activation in the intestinal epithelium triggers adenoma formation and represents a necessary, though insufficient, step for malignant transformation. Somatic mutations in *KRAS*, *BRAF*, *TP53*, and *SMAD4* usually underlie the further progression of the benign tumour into locally invasive adenocarcinoma and metastasis at distant organ sites [1]. Mutations in the endogenous mouse *Apc* gene also lead to intestinal polyp formation, though mainly located in the upper GI tract. Notably, mouse *Apc*-driven intestinal adenomas do not spontaneously accumulate *Kras* or *Tp53* somatic mutations and, accordingly, very rarely progress to adenocarcinomas [12].

In our laboratory, we have generated the *Apc* ^1638N/+^ mouse model encoding for a hypomorphic *Apc* mutation resulting in few (5–6) upper GI adenomas, longer survival, and an increased chance of spontaneous malignant transformation though only in animals older than 1 year [10], [11], [12]. In contrast, compound heterozygous *Apc* ^1638N/+^/*KRAS* ^V12G^ mice are characterized by increased tumour multiplicity (∼10 fold) and accelerated malignant progression with the majority of lesions being locally invasive adenocarcinomas [14]. Notably, oncogenic activation of *KRAS*/*Kras* on its own is insufficient to initiate intestinal tumorigenesis [25], if not with late onset and upon somatic inactivation of the *Tp53* gene [13]. Hence, the dramatic phenotypic differences brought about by oncogenic activation of the *KRAS* gene in compound *Apc* ^1638N/+^/*KRAS* ^V12G^ mice results from its synergistic action in promoting canonical Wnt signalling, as shown by the increase in TOP-Flash reporter activity and in the number of tumour cells earmarked by nuclear β-catenin accumulation [14].

The observed relative increase in the number of *Apc* ^1638N/+^/*KRAS* ^V12G^ tumour cells earmarked by nuclear β-catenin is of importance in view of the so-called “β-catenin paradox” [7]: notwithstanding the fact that loss of *APC* function is common to all tumour cells and is predicted to result in the intracellular and nuclear accumulation of β-catenin, this is only observed in a minority of cancer cells undergoing an epithelial-to-mesenchymal transition (EMT) and non-randomly distributed at the invasive front of the tumour mass [4], [5] (see also Fig. 2a). This observation led to the hypothesis according to which nuclear β-catenin earmarks stem-like intestinal tumour cells with higher Wnt signalling activity capable of detaching from the primary mass and efficiently disseminate and home in distal, vital organs [6], [19], [26].

Here, we attempted the prospective purification of cancer stem cells (CSCs) from *Apc* ^1638N/+^/*KRAS* ^V12G^ intestinal tumours. Notably, the presence of tumour-initiating cells, could not be demonstrated in *Apc* ^1638N/+^ tumours. According to the operational CSC definition, namely their capacity to form tumours upon limiting dilution transplantation into recipient mice, *bona fide* CSCs are either absent or extremely rare (>10^−6^) in the intestinal lesions characteristic of *Apc* ^1638N/+^ mice. In contrast, the malignant transformation and accelerated adenoma-carcinoma sequence brought about by the gut-specific expression of oncogenic *KRAS* result in the establishment of a subpopulation of tumour-initiating cells (estimated as 1 in approx. 7×10^4^ bulk tumour cells). The observed lack of grafting potential by cells derived from *Apc* ^1638N/+^ tumours is not surprising in view of previous studies showing that both established adenoma cell lines and primary human adenomas cannot form tumours even when injected at very high multiplicities (∼10^7^) [27], [28], [29], [30]. This is also in agreement with the recent study by C. Blanpain and colleagues showing that benign papillomas of the skin form secondary tumours at very low frequencies exclusively when transplanted together with stroma cells [31]. Of note, although the tumours here utilized for the transplantation and expression profiling analyses were not analysed by histopathology to maximize the number of cells available for these assays, it is safe to assume that the employed *Apc* ^1638N/+^ tumours were adenomas, whereas those from *Apc* ^1638N/+^/*KRAS* ^V12G^ animals were adenocarcinomas. This assumption is based on our own 10-yrs long experience with the histo-pathological analysis of the intestinal tumours found in these inbred models, in agreement with the original descriptions [10], [11], [12], [14].

Overall, these results call for a revision of the current operational definition of CSCs, especially when dealing with benign lesions where the alleged subpopulation of stem-like tumour cells are unlikely to form tumours upon transplantation in immune-deficient animals. Alternatively, it is also plausible to think that benign tumours do not encompass a *bona fide* subpopulation of CSCs and that the true cancer stem cell properties are acquired only upon malignant transformation.

CSCs enrichment (approx. 20–25 fold) was observed in the CD24^hi^CD29^+^ subpopulation (here also referred to as P3) of tumour cells from *Apc* ^1638N/+^/*KRAS* ^V12G^ mice. Notably, P3 cells are characterized by increased intracellular β-catenin accumulation thus indicating enhanced Wnt signalling activity and validating previously proposed models [6], [7]. These results are in apparent contradiction with a recent publication showing that oncogenic *Kras* activation does not result in the expansion of the intestinal stem cell pool [25]. However, these observations were made in an otherwise wild type genetic background (e.g. *Apc*-proficient) rather than in the context of constitutive Wnt activation resulting from loss of *Apc* gene function. As previously shown, *Apc* and *KRAS* mutations appear to be synergistic in enhancing Wnt signalling levels [14] and as such result in the expansion of the CSC compartment (earmarked by β-catenin intracellular accumulation) as shown here.

The CSCs concept [3] postulates that tumour masses are organized in a hierarchical fashion with a subpopulation of stem-like tumour cells endowed with self-renewal and differentiation capacity which underlie tumour maintenance and growth. Subpopulations of cancer cells enriched in CSCs have been isolated from the majority of tumour types, including colon cancer [15], [16], [17], [18]. However, apart from their capacity to recapitulate tumorigenesis when transplanted at limiting dilutions in immune-deficient mice, CSCs need to be further characterized by additional assays to test their self-renewal and differentiation capacity. As shown here, the CSCs enriched in the P3 subpopulation of *Apc* ^1638N/+^/*KRAS* ^V12G^ intestinal tumours are also characterized by self-renewal and differentiation: the relative size of the CSC subpopulation remains constant in serially transplanted tumours and the resulting malignant growths encompass several differentiated cell types (enterocytes, Goblet and Paneth cells) similar to the primary lesions. Notably, an apparent increase in the relative presence of intestinal differentiation lineages, namely Goblet, Paneth, and entero-endocrine cells, was noted among the serially transplanted intestinal tumours when compared with the primary *Apc* ^1638N/+^/*KRAS* ^V12G^ lesions. However, this was not reflected by a corresponding increase in the size of the CD29/CD24 tumor subpopulations as observed by FACS (Figure S3). Also, as these transplantation assays were subcutaneous and not orthotopic, it is likely that fluctuations in the incidence of specific lineages results from the influence of the host microenvironment on the CSCs’ self-renewal and differentiation rates.

Expression profiling analysis of CD24^hi^CD29^+^ cells from *Apc* ^1638N/+^ and *Apc* ^1638N/+^/*KRAS* ^V12G^ tumours in comparison with bulk and more differentiated tumour cells revealed several biological processes likely to play functional roles in cancer stemness. Gene ontology (GO) analysis [20] of the P3 signature revealed a broad spectrum of biological and molecular functions (Table S6). Of note, protein (mainly growth factors) binding activity and extracellular matrix (ECM) structural constituents seems to characterize the expression profile of the P3 subpopulation of tumour cells. Whereas the former (growth factor binding) may represent the main mechanism underlying the activity of specific signalling pathways in CSCs, ECM components are likely to play a rate/limiting supportive role in the regulation of essential stem cell properties cells (eg, self-renewal, multipotency, proliferation, and differentiation). Stem cell niches are known to be regulated by direct and paracrine interactions with supporting cells and the extracellular matrix. Likewise, cancer-associated fibroblasts and other (e.g. inflammatory) cellular components of the tumour microenvironment modulate the CSC niche not only by releasing specific growth factors and cytokines but also through the synthesis of modified ECM components rarely found in normal tissues [32]. Nevertheless, one should not forget that the P3 subpopulation is enriched in stem-like tumour cells and by no means represents purified CSCs. Hence, bioinformatic analysis of the corresponding gene profiles might be confounded by the presence of different cellular components which do not make integral part of the CSC subpopulation and should be interpreted with caution. Recently, a signature that predicts disease relapse in colon cancer in man was developed based on mouse intestinal stem cells (ISC) expressing high levels of the *Lgr5* and *EphB2* genes [18]. Notably, comparison between the P3 and *EphB2* ^hi^ and *Lgr5* ^hi^ signatures revealed no major overlap (7 and 13 genes respectively, among which *Lgr5* but not *EphB2*).

Of note, several Paneth cell specific genes including defensins (*Defa5*, *Defa 21/22*, *Defa24*), secretory phospholipase A2 (*Pla2g2a*), *Fzd5* and matrix metallopeptidase 7 (*Mmp7*) are differentially expressed in the P3 population [33]. On one hand this is expected as the P3 (Lin^−^CD24^hi^CD29^+^) FACS gate is likely to overlap with the CD24^hi^SSC^hi^ sorting gate previously employed to enrich for Paneth cells from normal small intestinal epithelial cells [34], [35]. Nevertheless, this observation is of potential interest in view of the recently elucidated dual role of Paneth cells both as niche cells (supporting *Lgr5* ^+^ stem cells) [34], [35], and as quiescent stem cells [35], [36]. Given the concomitant *Lgr5* upregulation in P3 cells, it is plausible that tumour-specific Paneth cells constitute the niche for *Lgr5* ^+^ CSCs in *Apc* ^1638N/+^/*KRAS* ^V12G^ adenocarcinomas [37]. Alternatively, quiescent (Paneth cell precursor) tumour stem cells may provide a source of actively cycling *Lgr5^+^* CSCs whose function is then further supported by more mature Paneth cells.

Notably, the *Apc*- and *Apc*/*KRAS*-mutant genotypes could not be resolved by HCA and PCA which does not allow us to pinpoint qualitative differences between benign (*Apc* ^1638N/+^) and more malignant (*Apc* ^1638N/+^/*KRAS* ^V12G^) CSCs. This is possibly due to the relatively limited number of *Apc* ^1638N/+^ vs. *Apc* ^1638N/+^/*KRAS* ^V12G^ tumour samples (n = 5 for each group) employed for the comparative expression profiling analysis, insufficient to highlight the allegedly more subtle differences between the two CSCs genotypes. However, both in the HCA and PCA a trend is apparent where the P1+2 and P3 tumour cell subpopulations derived from *Apc* ^1638N/+^ and *Apc* ^1638N/+^/*KRAS* ^V12G^ tumours appear to cluster away from each other. In the future, the use of more tumour samples and possibly the profiling of microdissected tumour cells earmarked by nuclear β-catenin accumulation from the two genotypes will shed more light on the more qualitative differences between benign vs. malignant neoplastic stem cells.

Overall, our results show that cancer stemness in *Apc*-driven intestinal tumorigenesis is underlined by an increased Wnt/β-catenin signalling activity that is apparently decreased in more differentiated tumour cells. As previously [7] and here reported (Figure 2), in both human (colon) and mouse (upper GI tract) *APC*-driven intestinal cancers cells with intracellular β-catenin accumulation are clustered around the invasive front, thus suggesting that factors secreted from the stromal microenvironment are likely to play a role in the enhancement of Wnt signalling activity and the maintenance of stem-like cancer cells. Also, the differential upregulation in the P3 subpopulation of markers of both cycling stem cells (*Lgr5*) and Paneth cells (defensins, matrix metalloproteases, and phospholipases) suggest that a niche similar to what described for the normal intestinal crypt of the mouse is present within adenocarcinomas. Further purification and (epi)genetic profiling of the individual components of the CSC niche are necessary to elucidate the cellular and molecular mechanisms underlying cancer stemness in the digestive tract.

## Materials and Methods

### Ethics Statement

This study has been approved by the stichting Dier Experimenten Commissie (DEC), approval number EUR1383 and 2351.

During all mouse experimental interventions, animals have been anesthetized to minimize pain and stress and maintained at body temperature. Pain treatment for surgery has been done using Temgesic. According to the criteria as described by the “*Code of Practice Dierproeven in het Kankeronderzoek*”, regular controls by animal care personnel and our own scientist have been conducted throughout the study to minimize suffering and discomfort due to the intestinal and subcutaneous malignancies with coinciding anaemia and weight loss.

### Tumor Harvest and Dissociation

Tumors were harvested from *Apc^1638N^* ^/+^ and *Apc* ^1638N/+^/*KRAS* ^V12G^ mice aged 6 to 8 months. Tumors from individual animals were pooled, minced with a razor blade, and suspended into 10 ml digestion medium (RPMI, EGF 5 ng ml^−1,^ Hydrocortisone 50 ng ml^−1^, Insulin/Transferrin) supplemented with 10% foetal calf serum and containing 4 mg ml^−1^ collagenase A (#11088793001, Roche), 0.1 mg ml^−1^ Dispase II (#165859, Roche) and 50 µg ml^−1^ DNaseI (#DN-25, Sigma). Digestions were conducted for 2 h at 37°C, 5% CO_2_. The final cell suspension was filtrated through a 70- µm cell strainer (BD) and stained with antibodies.

### Cell Labelling, Flow Cytometry and Sorting

Immunostaining was performed in PBS supplemented with 2% bovine calf serum at 4°C. Antibodies employed for labelling of extracellular epitopes were: Biotin-conjugated rat anti-mouse CD31 (#553371, PECAM-1 Monoclonal, BD, 1∶100), Biotin-conjugated rat anti-mouse CD45-Biotin (#553077, BD, 1∶200), Biotin-conjugated rat anti-mouse TER119-Biotin (#553672,BD, 1/100), Streptavidin-PerCP-Cy5.5 conjugate (#551419, BD, 1∶1000), APC conjugated anti-mouse CD24 (#101813, Biolegend, 1∶250), Phycoerythrin (PE) conjugated Hamster monoclonal to CD29/integrin beta 1 (#ab36219, Abcam, 1∶25), FITC conjugated rat monoclonal anti CD44 antibodies (#553133, BD, 1∶200), mouse monoclonal anti L1CAM antibodies (#ab24345, Abcam, 1∶300)+secondary anti mouse-FITC antibodies, FITC conjugated mouse monoclonal anti CD97 antibodies (#ab23490, Abcam, 1∶10). Antibody incubations were performed for 30 min. at 4°C. For live/dead discrimination, cells were resuspended in 1 µg/ml Hoechst 33258 (#H3569, Invitrogen). Cell analysis and sorting were performed with a FACSAriaTM (BD Biosciences), equipped with a 100 µm nozzle. Hoechst fluorescence was detected using 405 excitation and a BP450/40 emission filter, FITC fluorescence was detected using 488 nm excitation and LP502+ BP 530/30 emission filters, PE fluorescence was detected using 488 nm excitation and LP556+ BP 575/26 emission filters, PerCP-Cy5.5 fluorescence was detected using 488 nm excitation and LP655+ BP 695/40 emission filters, APC fluorescence was detected using 633 nm excitation and a BP660/20 emission filter.

### Expression Profiling Analysis

Intestinal tumors from 3 *Apc* ^1638N/+^ mice and 5 *Apc* ^1638N/+^/*KRAS* ^V12G^ animals were employed for expression profiling by oligonucleotide arrays. For each mouse, tumors were pooled and digested as described above to allow sorting by FACS. From each of the Lin^−^, Lin^−^CD24^hi^CD29^+^ and Lin^−^CD24^med^CD29^+^/CD24^lo^CD29^+^ (joined gate) populations, total RNA was isolated from 10^4^ sorted cells by RNAeasy Micro kit (QIAGEN) and quality controlled by RNA 6000 Pico and Nano LabChip kits (Agilent Technologies). Sample labelling, hybridization, staining and scanning were performed on the Affymetrix MOE430 plus2.0 microarray platform using the Two-cycle Labelling kit according to manufacturer’s instruction.

The raw profiling data are available under GEO number **GSE47772** and can be viewed at: http://www.ncbi.nlm.nih.gov/geo/query/acc.cgi?acc=GSE47772.

For additional Materials and Methods see Methods S1.

## Supporting Information

Figure S1 **qPCR-based validation of genes differentially expressed in the P3 population.** Intestinal tumors from 2 individual *Apc* ^1638N/+^ and 3 individual *Apc* ^1638N/+^/*KRAS* ^V12G^ animals were digested to single cell suspensions and FACSorted for isolation of 10,000 cells from each of the Lin- (blue bars), P3 (green bars) and P1+P2 (red bars; joined gate) populations. Total RNA was isolated, converted into cDNA, and employed for 35 Taqman® assays corresponding to the genes listed in Supplementary Table 2. All values were normalized to the expression of the glyceraldehyde 3-phosphate dehydrogenase (*Gapdh*) house-keeping gene. Similar results were obtained with the β-actin gene (*Actb*) as reference.
(PDF)

Figure S2 **Representative H&E images from a tumor obtained by injecting Lin^−^ (bulk) cells from** ***Apc*** **^1638N/+^/** ***KRAS*** **^V12G^ intestinal tumours.**
(PDF)

Figure S3 **Relative size of the CD24/CD29 FACS subpopulations as observed in primary and serially transplanted tumors from** ***Apc*** **^1638N/+^/** ***KRAS*** **^V12G^ mice.** “Total tumors” refers to a total of 30 tumors analyzed by FACS in our laboratory between Augustus 2008 and December 2009, employed for purposes other than serial transplantations. “Parental tumors” represent the primary *Apc* ^1638N/+^/*KRAS* ^V12G^ lesions employed as source for the serial transplantations, here referred to as 1^st^ and 2^nd^ round tumors. Despite some nearly-significant fluctuations, the data indicate only minimal overall changes between primary tumors and serially transplanted ones.
(PDF)

Figure S4 **a.** Western blot representative of the β-catenin analysis of the FACSorted populations from *Apc* ^1638N/+^/*KRAS* ^V12G^ intestinal tumours. Both the antibodies directed total (green, upper panel) and actively signaling (de-phosphorylated; red, middle panel) β-catenin are shown. β-actin (lower panel, red) was employed as a loading control. Legend: M = molecular weight marker; 1. Lin^−^ (bulk); 2. Lin^−^CD24^−^CD29^−^; 3. Lin^−^CD24^+^; 4. Lin^−^CD29^+^; 5. Lin^−^P1+P2+P3; 6. Lin^−^P1; 7. Lin^−^P2; 8. Lin^−^P3. **b.** Western blot analysis of total β-catenin in primary *Apc* ^1638N/+^ intestinal tumours. The bars represents the quantification of the bands by scanning and analysing the western blot with the Odyssey scanner and after normalization with β-actin. When the anti-active β-catenin Ab was employed, hardly any signal could be detected in bulk and sorted *Apc* ^1638N/+^ intestinal tumour cells.
(TIF)

Table S1 Subcutaneous transplantation of tumour cells from *Apc* ^1638N/+^/*KRAS* ^V12G^ mice in immune-incompetent mice sorted with common CSC surface antigen markers.
(PDF)

Table S2 List of the 35 genes and corresponding Taqman® assays employed to validate the microarray data by qRT-PCR (see Supplementary Figure 2).
(PDF)

Table S3 P3 signature based on P3 vs. Lin^−^ comparison. ANOVA (3 ways) analysis led to an initial list of 746 differential expressed probsets (n = 602 non-redundant, annotated RefSeq Genes) with a FDR of 0.05 and ≥2 fold difference. RefSeq Transcript IDs, Probeset IDs and the lowest corresponding P-value (P3 vs Lin^−^) are listed. Uploading/mapping this list in Biobase-Explain resulted in 574 annotated genes.
(XLS)

Table S4 P3 signature based on P3 vs. P1+P2 comparison (n = 808 annotated genes). ANOVA (3 ways) analysis led to an initial list of 1062 differential expressed probsets [851 non-redundant, annotated RefSeq genes) with a FDR of 0.05 and ≥2 fold difference. RefSeq Transcript IDs, Probeset IDs and the lowest corresponding p-value (P3 vs. P1+P2) are listed. Uploading/mapping this list in Biobase-Explain resulted in 808 annotated genes.
(XLS)

Table S5 P3 signature (intersection between P3 vs. Lin- and P3 vs. P1+P2 (n = 462 annotated genes). ANOVA (3 ways) analysis led to an initial list of 1062 differential expressed probesets (851 non-redundant, annotated RefSeq genes) with a FDR of 0.05 and ≥2-fold difference. Gene Symbol, Gene Title, RefSeq Transcript IDs, Probeset IDs and the lowest corresponding P-values (P3 vs. Lin- and P3 vs. P1+P2) are listed. Uploading/mapping this list in Biobase-Explain resulted in 462 annotated genes.
(XLS)

Table S6 Gene Ontology (GO) Analysis of the P3/intersection signature (see Supp. Table 5). Within Biobase/Explain, the Functional Analysis module enables to explore statistically overrepresented and underrepresented groups in a data set according to Gene Ontology classification (19). Matched terms of the selected Function category are given in each row of the output table. The table presents (from left to right) the GO identifier, Gene symbols, the GO term description, the number of hits from the input set to the ontology group, the size of the group in the database, expected hits, and the P-value of the observation.
(XLS)

Methods S1 **See Supplementary Materials and Methods file.**
(DOC)
