# Supplementary figures and images for: Cancer Stemness in Apc- vs. Apc/KRAS-Driven Intestinal Tumorigenesis
Source: PLoS One. 2013 Sep 17;8(9):e73872. doi: 10.1371/journal.pone.0073872 (PMC3775784; doi:10.1371/journal.pone.0073872)

Supplementary Figure 1

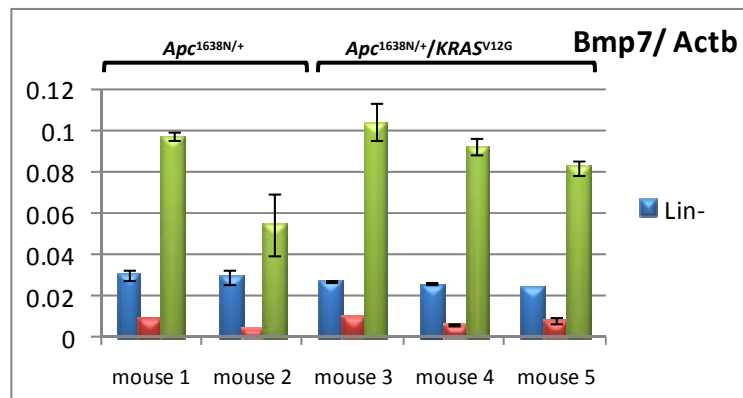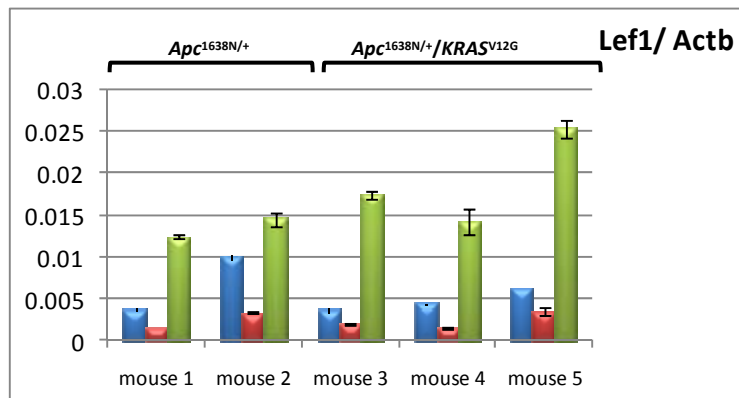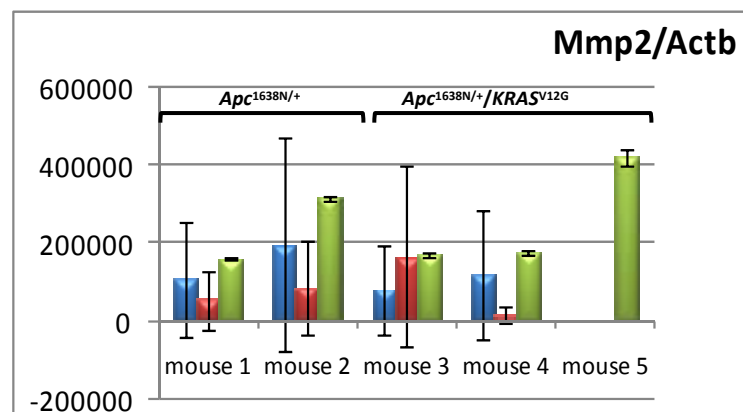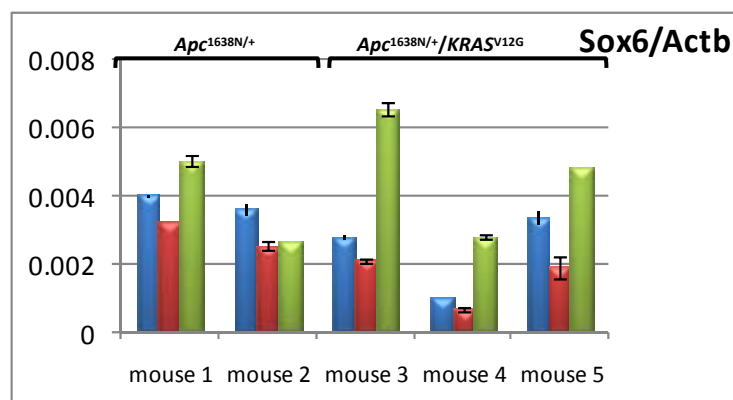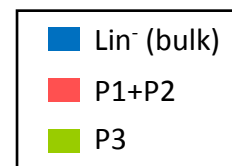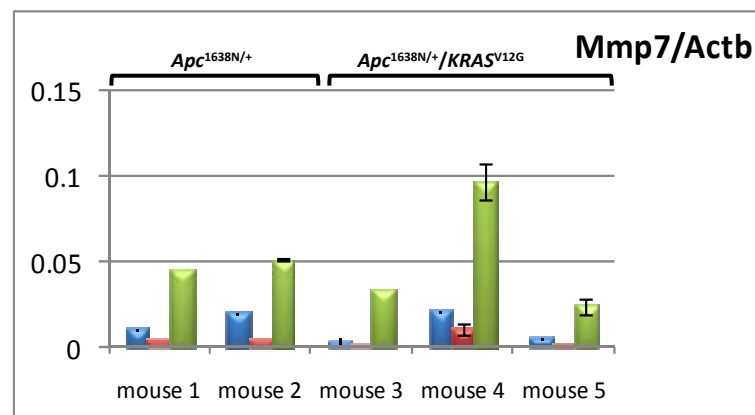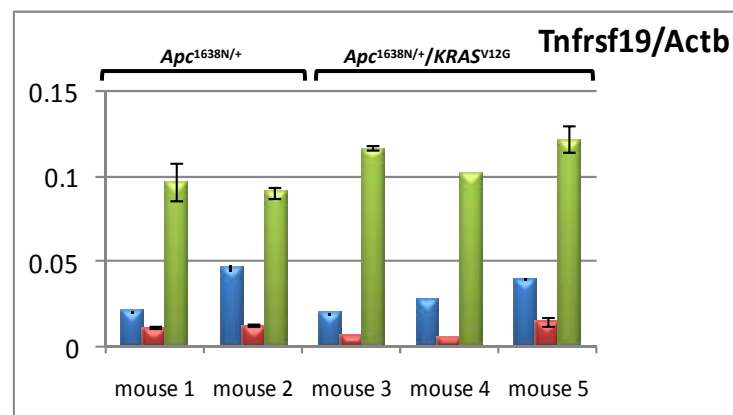

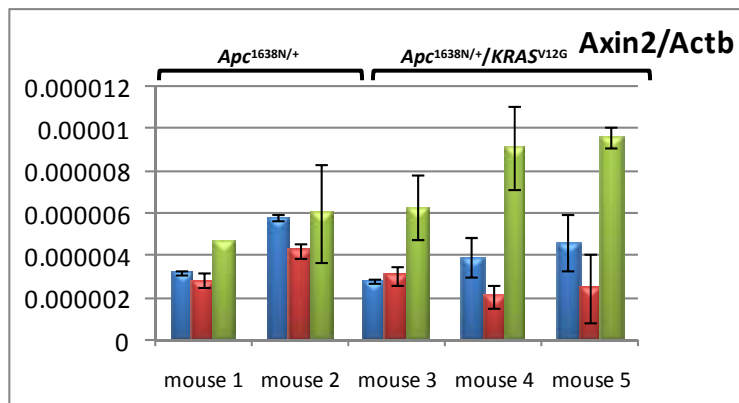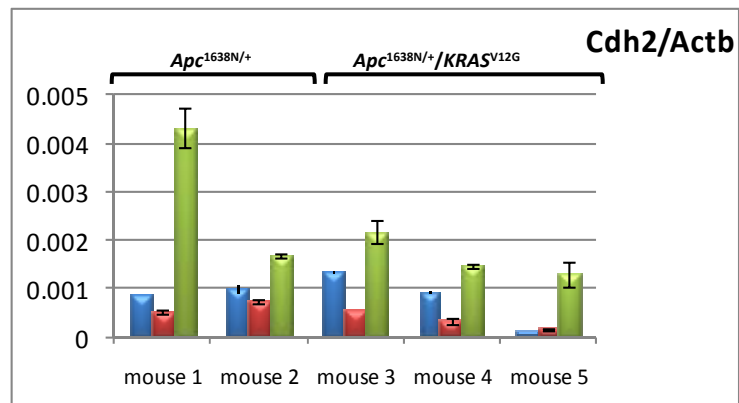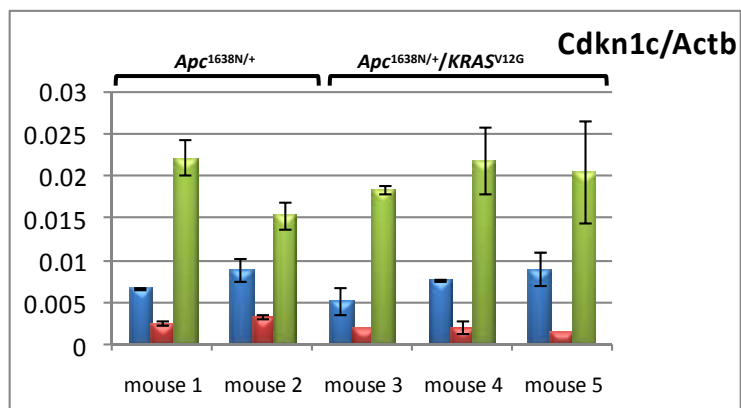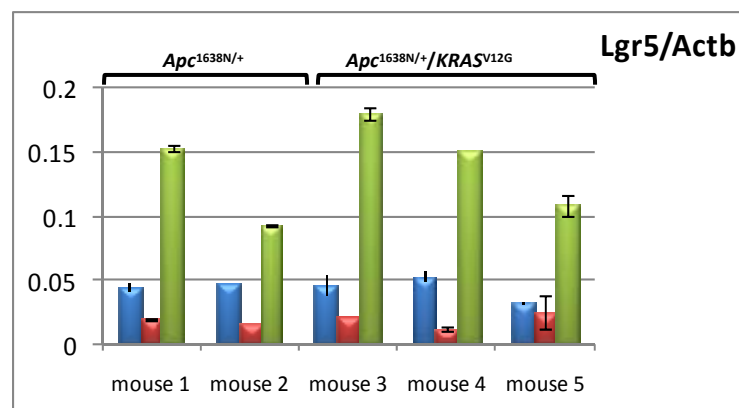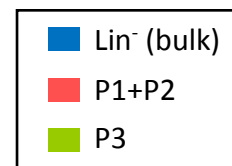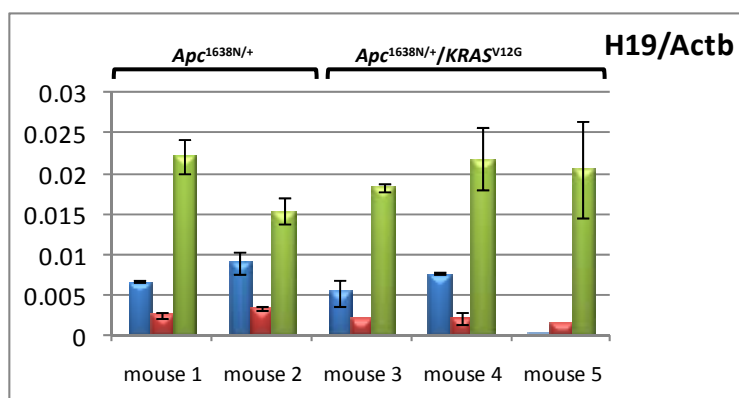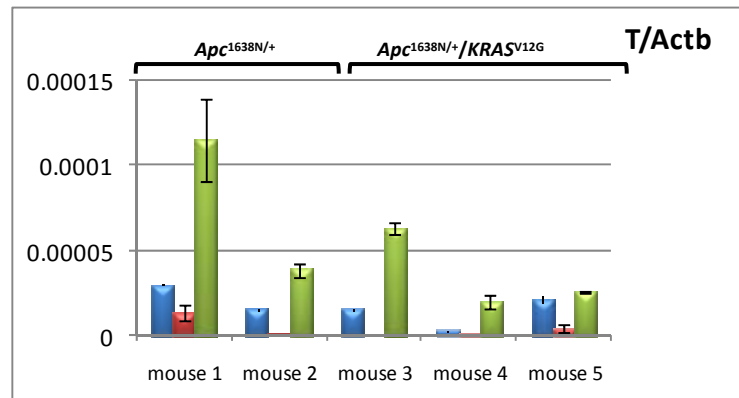

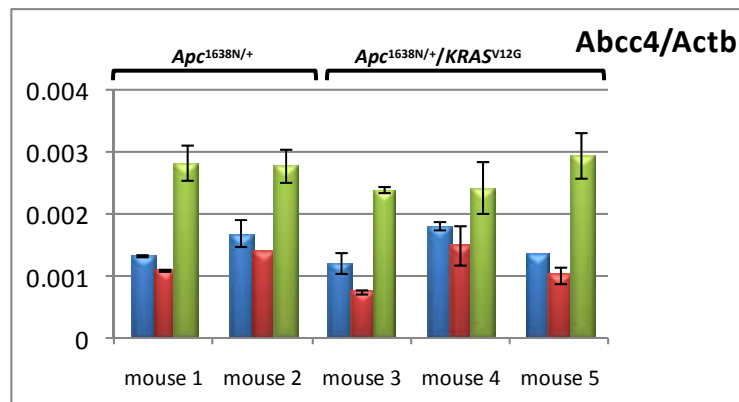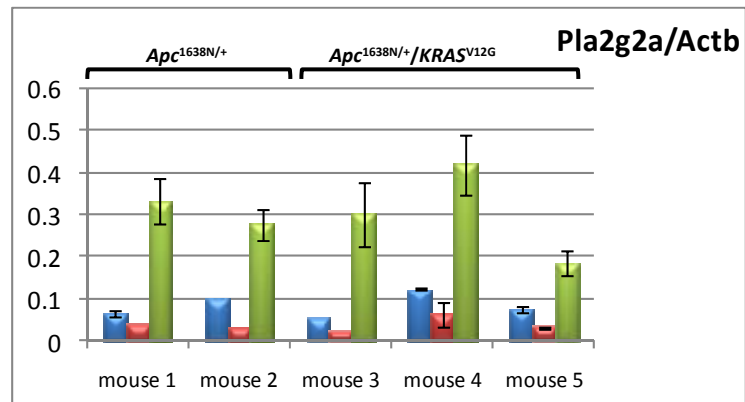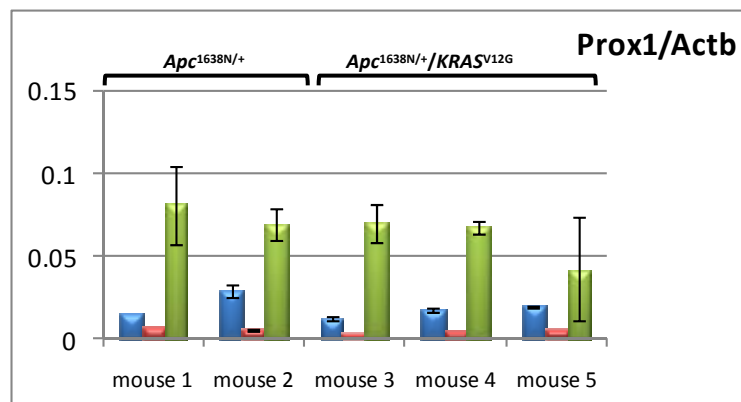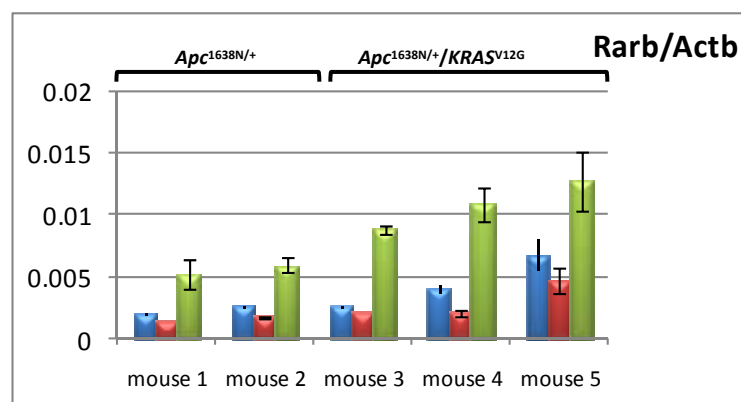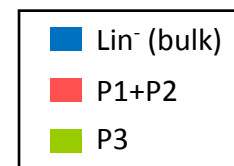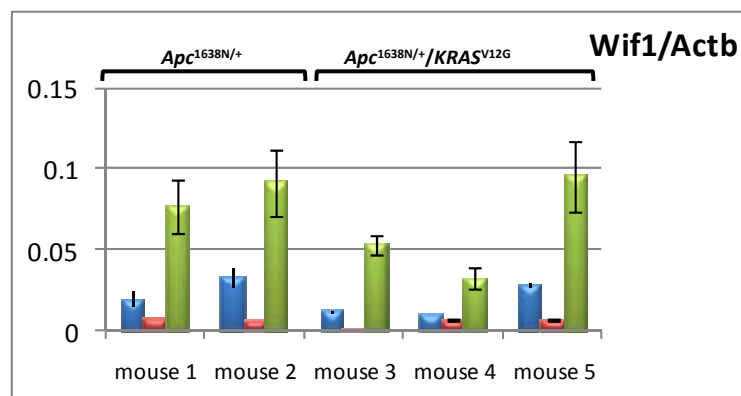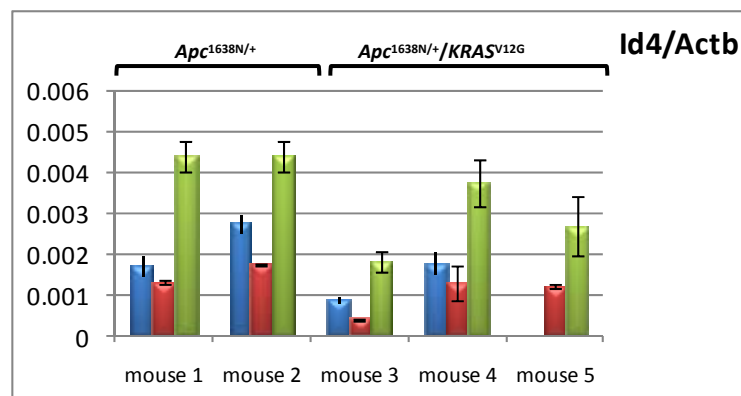

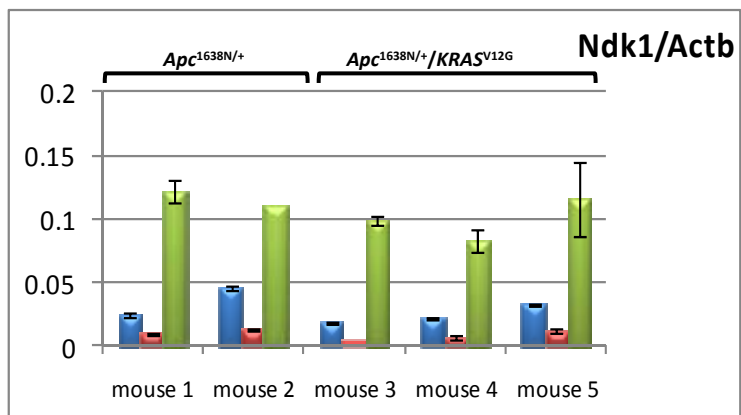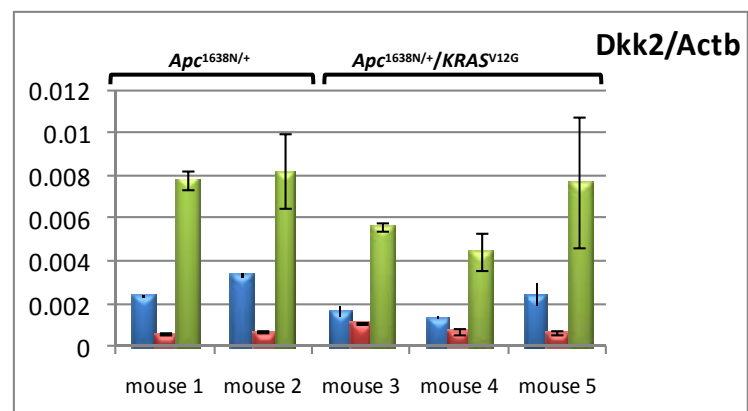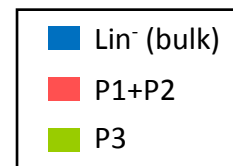

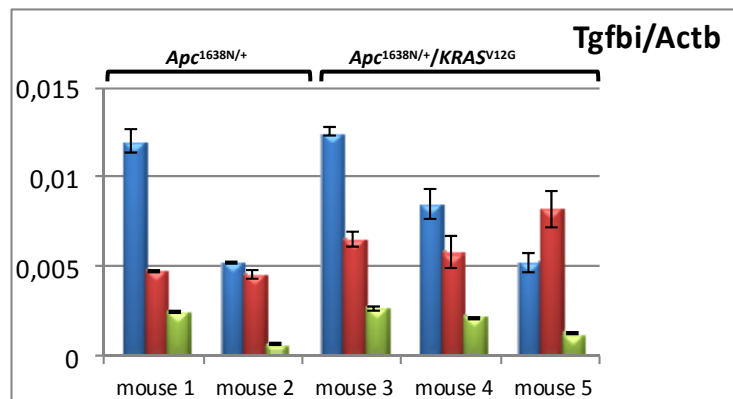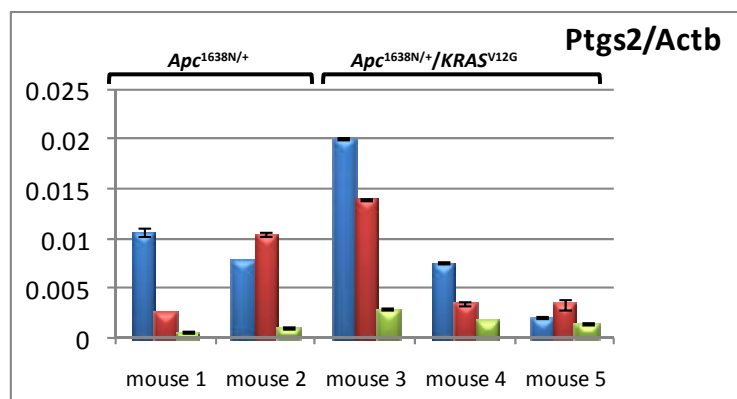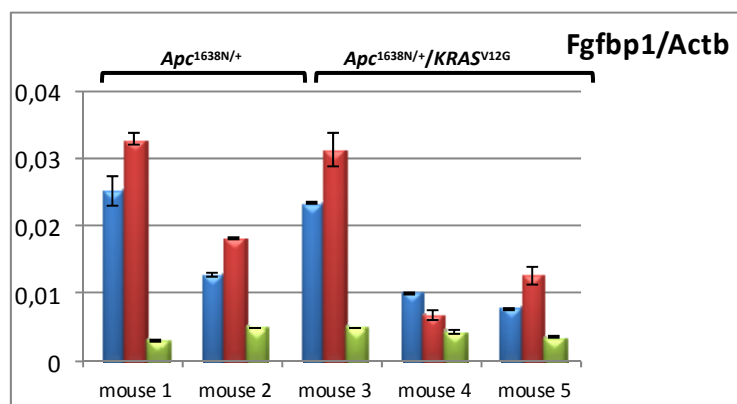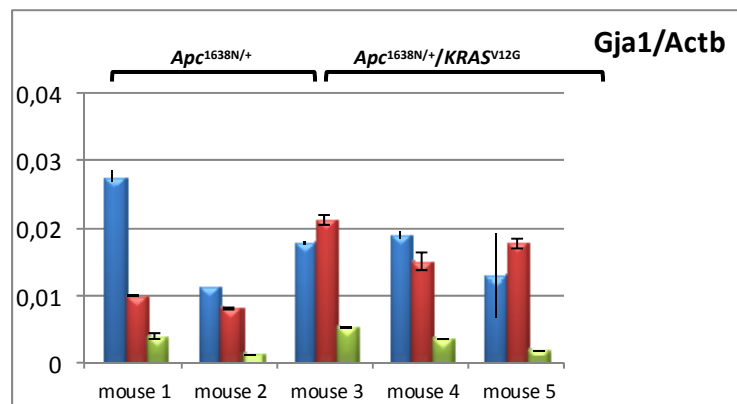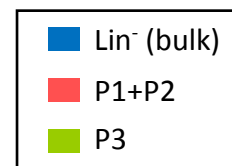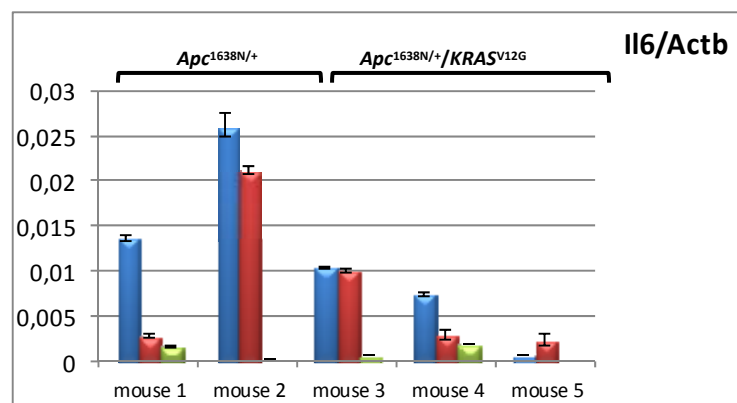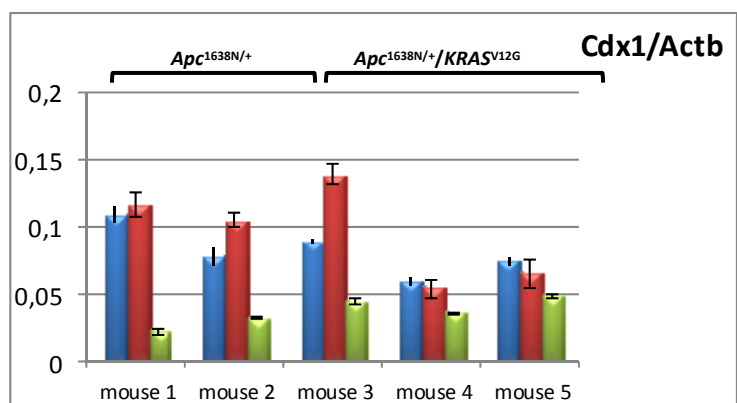

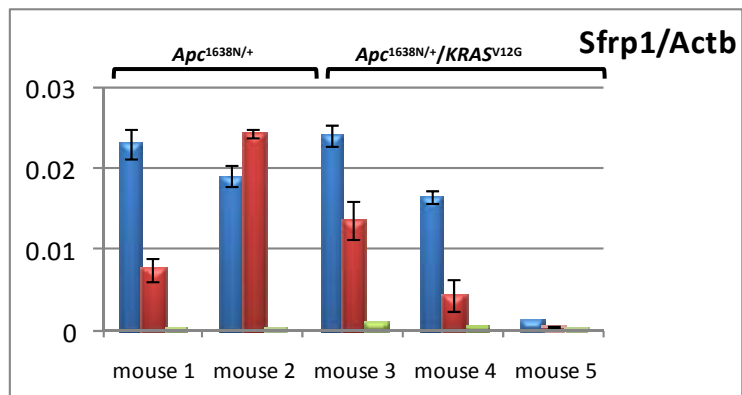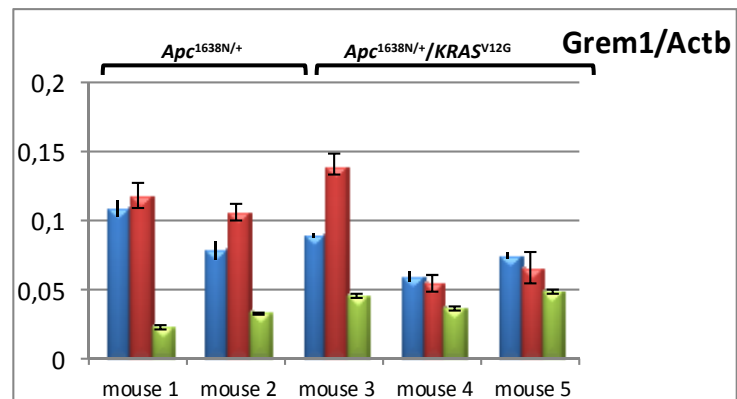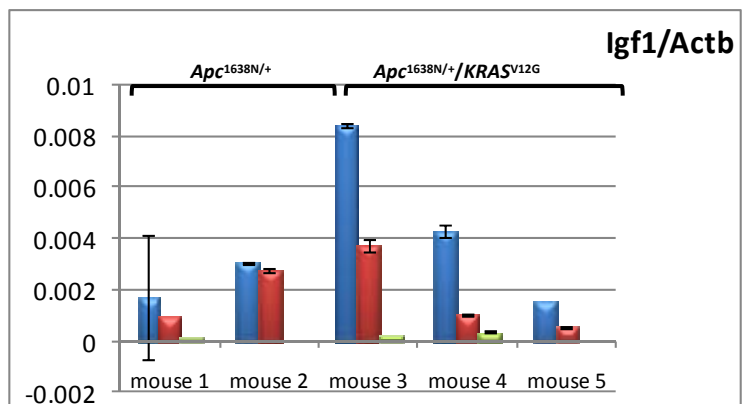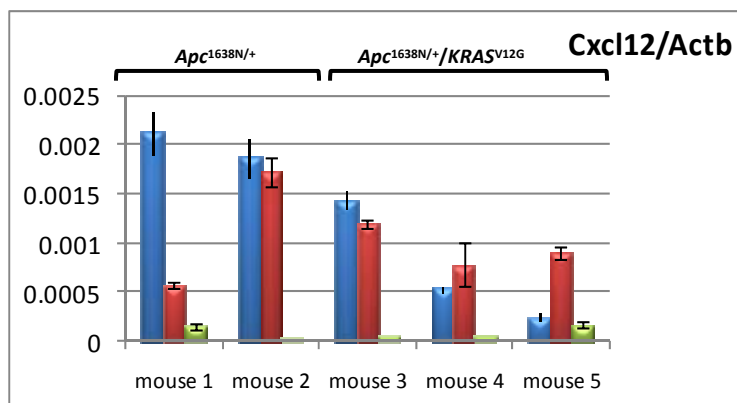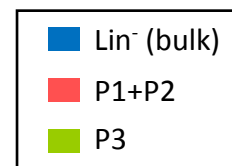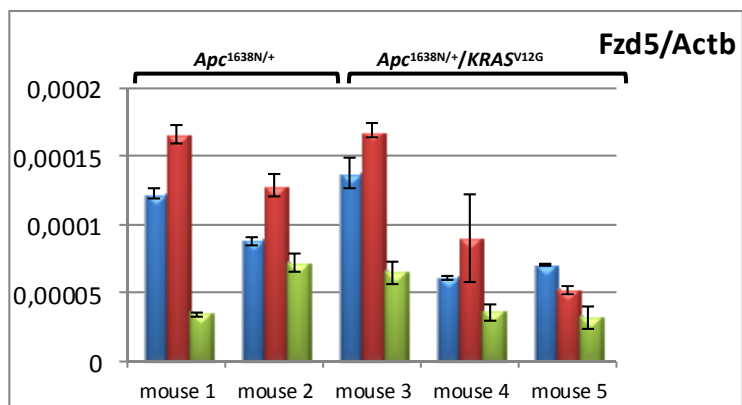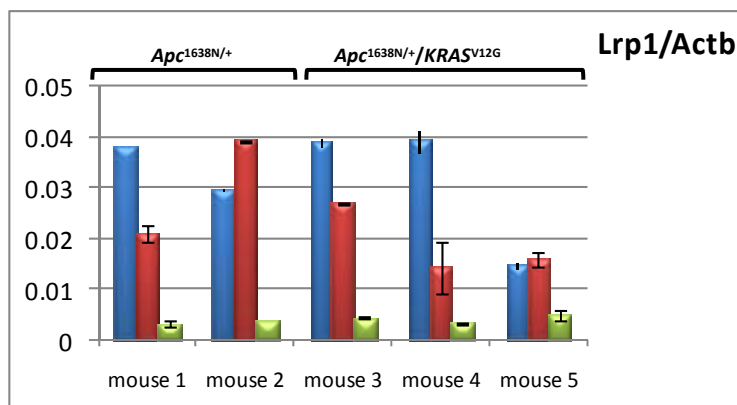

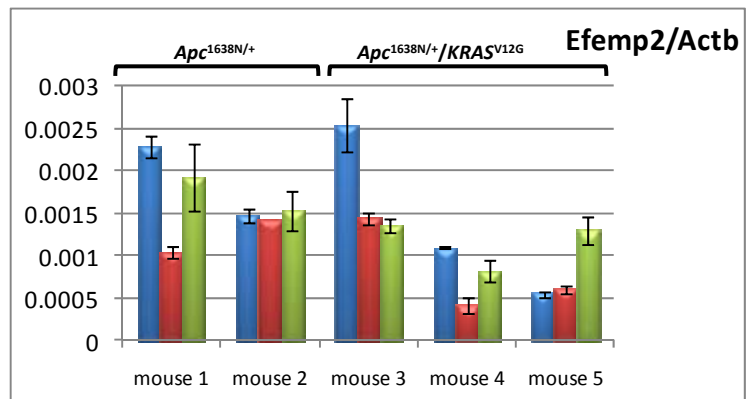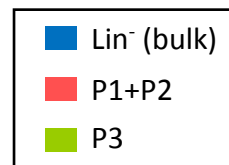

Supplement: Figure S1 — qPCR-based validation of genes differentially expressed in the P3 population. Intestinal tumors from 2 individual Apc 1638N/+ and 3 individual Apc 1638N/+/KRAS V12G animals were digested to single cell suspensions and FACSorted for isolation of 10,000 cells from each of the Lin- (blue bars), P3 (green bars) and P1+P2 (red bars; joined gate) populations. Total RNA was isolated, converted into cDNA, and employed for 35 Taqman® assays corresponding to the genes listed in Supplementary Table 2. All values were normalized to the expression of the glyceraldehyde 3-phosphate dehydrogenase (Gapdh) house-keeping gene. Similar results were obtained with the β-actin gene (Actb) as reference. (PDF) [file pone.0073872.s001.pdf]

Supplementary Figure 2

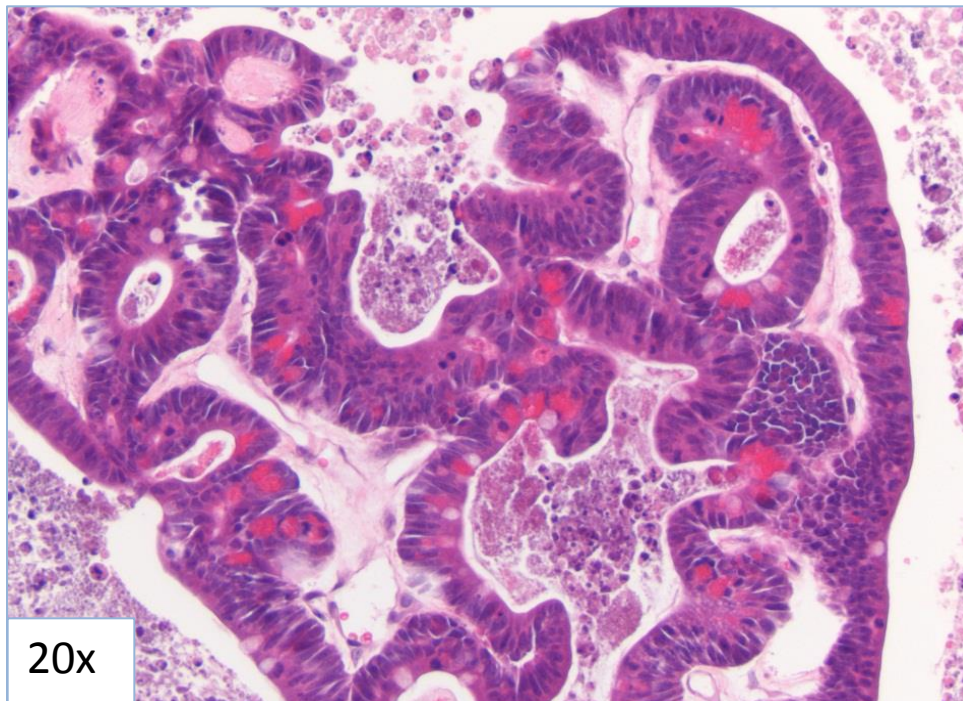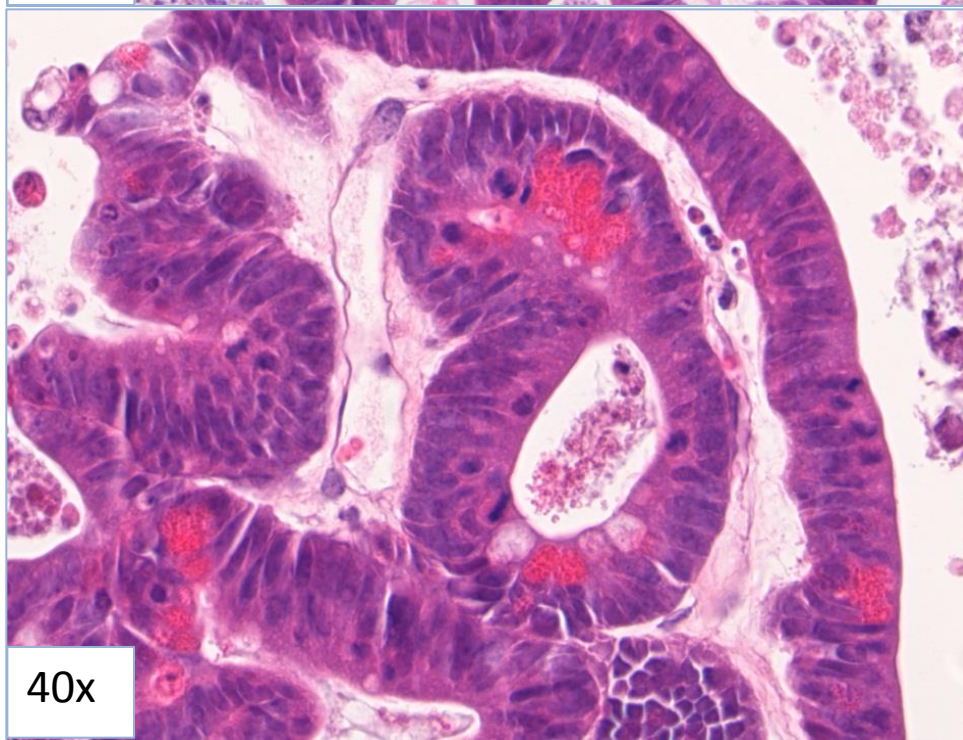

Supplement: Figure S2 — Representative H&E images from a tumor obtained by injecting Lin− (bulk) cells from Apc 1638N/+/ KRAS V12G intestinal tumours. (PDF) [file pone.0073872.s002.pdf]

**Supplementary Figure 3**

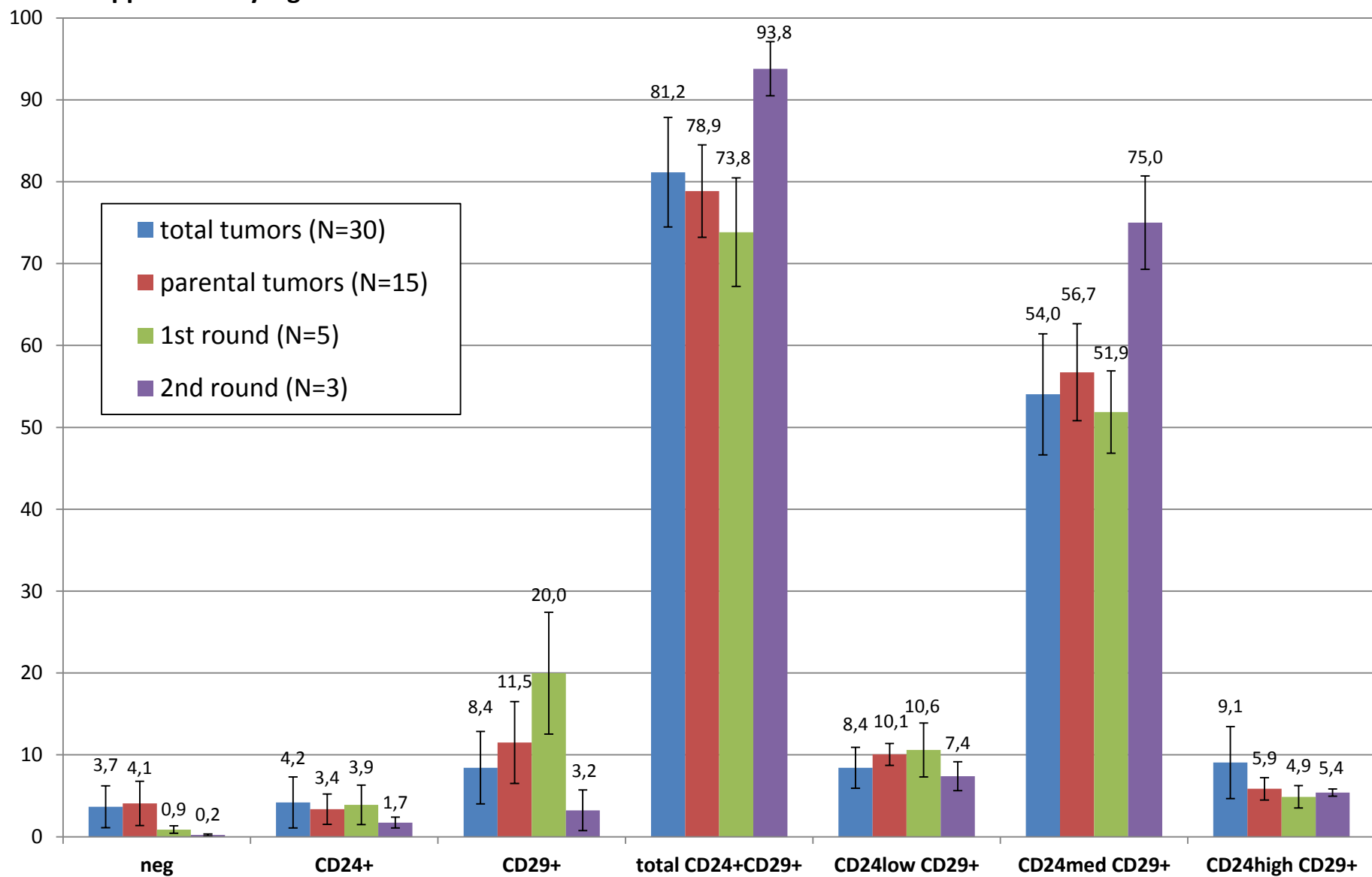

Supplement: Figure S3 — Relative size of the CD24/CD29 FACS subpopulations as observed in primary and serially transplanted tumors from Apc 1638N/+/ KRAS V12G mice. “Total tumors” refers to a total of 30 tumors analyzed by FACS in our laboratory between Augustus 2008 and December 2009, employed for purposes other than serial transplantations. “Parental tumors” represent the primary Apc 1638N/+/KRAS V12G lesions employed as source for the serial transplantations, here referred to as 1st and 2nd round tumors. Despite some nearly-significant fluctuations, the data indicate only minimal overall changes between primary tumors and serially transplanted ones. (PDF) [file pone.0073872.s003.pdf]

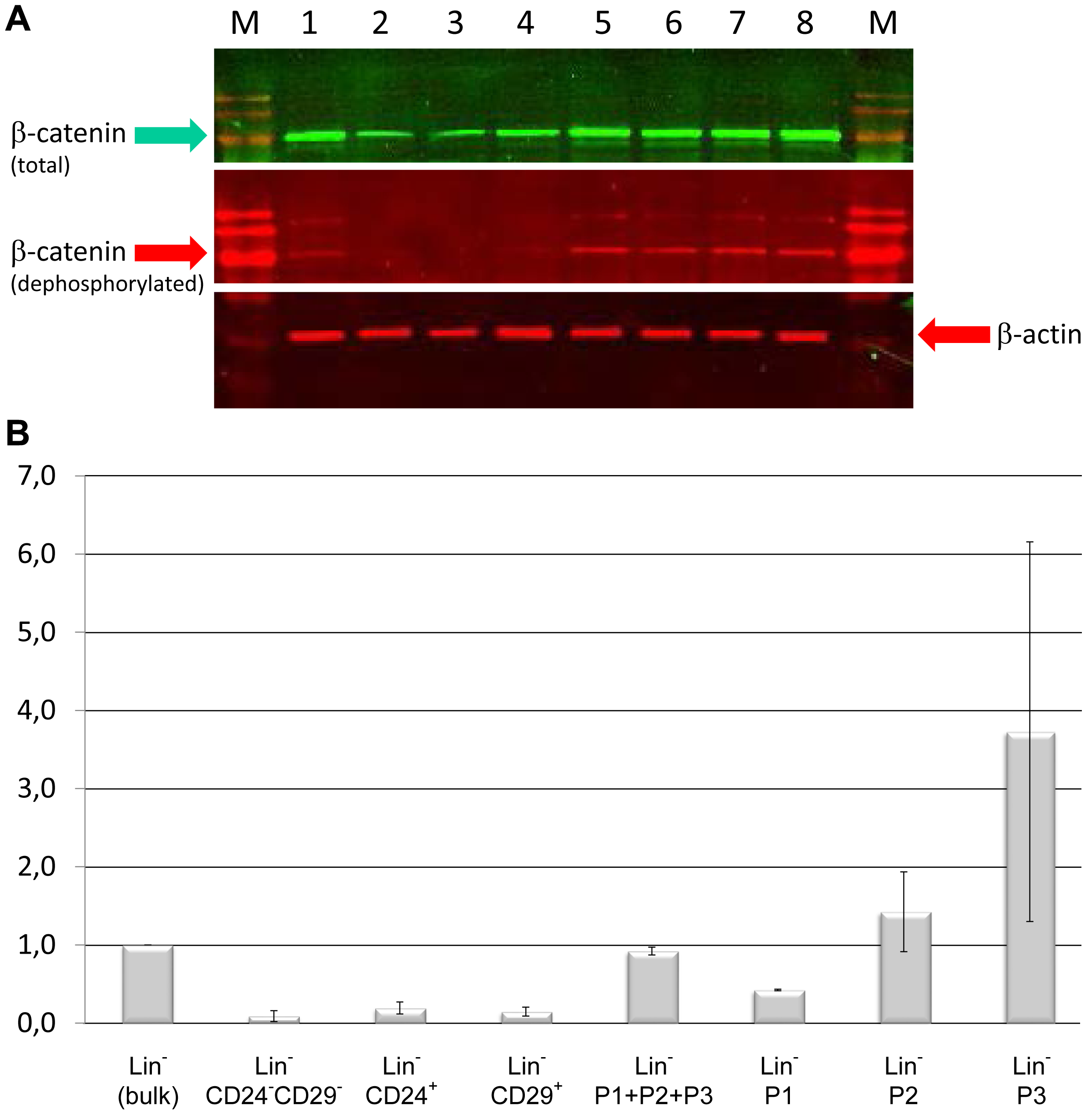

Supplement: Figure S4 — a. Western blot representative of the β-catenin analysis of the FACSorted populations from Apc 1638N/+/KRAS V12G intestinal tumours. Both the antibodies directed total (green, upper panel) and actively signaling (de-phosphorylated; red, middle panel) β-catenin are shown. β-actin (lower panel, red) was employed as a loading control. Legend: M = molecular weight marker; 1. Lin− (bulk); 2. Lin−CD24−CD29−; 3. Lin−CD24+; 4. Lin−CD29+; 5. Lin−P1+P2+P3; 6. Lin−P1; 7. Lin−P2; 8. Lin−P3. b. Western blot analysis of total β-catenin in primary Apc 1638N/+ intestinal tumours. The bars represents the quantification of the bands by scanning and analysing the western blot with the Odyssey scanner and after normalization with β-actin. When the anti-active β-catenin Ab was employed, hardly any signal could be detected in bulk and sorted Apc 1638N/+ intestinal tumour cells. (TIF) [file pone.0073872.s004.tif]
