# Supplementary material for: Cancer Stemness in Apc- vs. Apc/KRAS-Driven Intestinal Tumorigenesis
Source: PLoS One. 2013 Sep 17;8(9):e73872. doi: 10.1371/journal.pone.0073872 (PMC3775784; doi:10.1371/journal.pone.0073872)
Supplement: Table S1 — Subcutaneous transplantation of tumour cells from Apc 1638N/+/KRAS V12G mice in immune-incompetent mice sorted with common CSC surface antigen markers. (PDF) [file pone.0073872.s005.pdf]

Supplementary Table 1. Subcutaneous transplantation of tumor cells from *Apc*$^{1638N/+}$/*KRAS*^{V12G} adenocarcinomas in immune-incompetent mice sorted with common CSC surface antigen markers.

Sorted tumor population	*No. of transplanted cells*	*Tumors in NODSCID*
Lin^{-} (bulk tumor cells)	5.0×10^{3}	3/48
$Lin^{-}CD44^{+}$	5.0×10^{3}	1/12
$Lin^{-}CD44^{-}$	1.5×10^{3}	0/4
$Lin^{-}CD24^{+}CD29^{+}CD44^{+}$	5.0×10^{3}	2/17
$Lin^{-}CD24^{+}CD29^{+}CD44^{-}$	1.5×10^{3}	2/17
$Lin^{-}CD97^{+}$	5.0×10^{3}	0/12
$Lin^{-}CD24^{+}CD29^{+}CD97^{+}$	1.5×10^{3}	0/17
$Lin^{-}CD24^{+}CD29^{+}CD97^{-}$	1.5×10^{3}	0/17
$Lin^{-}CD24^{+}CD29^{+}L1CAM^{+}$	1.5×10^{3}	0/4
$Lin^{-}CD24^{+}CD29^{+}L1CAM^{-}$	1.5×10^{3}	0/4
$Lin^{-}L1CAM^{+}$	1.5×10^{3}	0/8
$Lin^{-}L1CAM^{-}$	1.5×10^{3}	0/4
