# Supplementary material for: Cancer Stemness in Apc- vs. Apc/KRAS-Driven Intestinal Tumorigenesis
Source: PLoS One. 2013 Sep 17;8(9):e73872. doi: 10.1371/journal.pone.0073872 (PMC3775784; doi:10.1371/journal.pone.0073872)
Supplement: Table S2 — List of the 35 genes and corresponding Taqman® assays employed to validate the microarray data by qRT-PCR (see Supplementary Figure 2). (PDF) [file pone.0073872.s006.pdf]

**Supplementary Table 2**

| Gene Symbol | Gene Name | Assay ID |
|---|---|---|
| *Gapdh* | glyceraldehyde 3-phosphate dehydrogenase | Mm99999915_g1 |
| *Actb* | actin B | Mm00607939_s1 |
| *Axin2* | Axi1, Axil, Conductin | Mm00443610_m1 |
| *Bmp7* | bone morphogenetic protein 7 | Mm00432102_m1 |
| *Cdx1* | caudal type homeo box 1 | Mm00438172_m1 |
| *Fgfbp1* | fibroblast growth factor-binding protein 1 | Mm00456064_s1 |
| *Fzd5* | frizzled homolog 5 (Drosophila) | Mm03053323_s1 |
| *Gja1* | gap junction protein, alpha 1, | Mm00439105_m1 |
| *Igf1* | insulin-like growth factor 1 | Mm00439561_m1 |
| *Il6* | interleukin 6 | Mm00446190_m1 |
| *Lef1* | lymphoid enhancer binding factor 1 | Mm00550265_m1 |
| *Lgr5* | leucine rich repeat containing G protein coupled receptor 5 | Mm00438890_m1 |
| *Mmp2* | matrix metallopeptidase 2 | Mm00439508_m1 |
| *Mmp3* | matrix metallopeptidase 3 | Mm00440295_m1 |
| *Nrcam* | Neuronal cell adhesion molecule | Mm00663614_m1 |
| *Ptgs2* | prostaglandin-endoperoxide synthase 2 | Mm00478374_m1 |
| *Sox6* | SRY-box containing gene 6 | Mm00488393_m1 |
| *T* | brachyury | Mm00436877_m1 |
| *Tgfbi* | transforming growth factor, beta induced | Mm00493634_m1 |
| *Tnfrsf19* | tumor necrosis factor receptor superfamily, member 19 | Mm00443506_m1 |
| *Grem1* | gremlin 1 | Mm00488615_s1 |
| *Abcc4* | ATP-binding cassette, sub-family C (CFTR/MRP), member 4 | Mm01226380_m1 |
| *Cdh2* | Cadherin-2 | Mm00483213_m1 |
| *Cdkn1c* | cyclin-dependent kinase inhibitor 1C | Mm01272135_g1 |
| *Cxcl 12* | chemokine (C-X-C motif) ligand 12 | Mm00445552_m1 |
| *Dkk2* | dickkopf-related protein 2 | m00445025_m1 |
| *Efemp2* | EGF-containing fibulin-like extracellular matrix protein 2 | Mm00445429_m1 |
| *H19* | H19 fetal liver mRNA | Mm01156721_g1 |
| *Id4* | Inhibitor of DNA binding 4 | Mm00499701_m1 |
| *Lrp1* | low density lipoprotein receptor-related protein 1 | Mm00464608_m1 |
| *Ndk1* | nucleoside Diphosphate Kinase 1 | Mm00471902_m1 |
| *Pla2g2a* | phospholipase A2, group IIA (platelets, synovial fluid) | Mm00448160_m1 |
| *Prox1* | prospero-related homeobox 1 | Mm00435969_m1 |
| *Rarb* | retinoic acid receptor beta | Mm01319674_m1 |
| *Sfrp1* | secreted frizzled-related protein 1 | Mm00489161_m1 |
| *Wif1* | Wnt inhibitory factor 1 | Mm00442355_m1 |
| *Mmp7* | matrix metallopeptidase 7 | |
| | | |
| *Gapdh* | glyceraldehyde 3-phosphate dehydrogenase | Mm99999915_g1 |
| *Actb* | actin B | Mm00607939_s1 |
