# Supplementary material for: Cancer Stemness in Apc- vs. Apc/KRAS-Driven Intestinal Tumorigenesis
Source: PLoS One. 2013 Sep 17;8(9):e73872. doi: 10.1371/journal.pone.0073872 (PMC3775784; doi:10.1371/journal.pone.0073872)
Supplement: Methods S1 — See Supplementary Materials and Methods file. (DOC) [file pone.0073872.s011.doc]

**Supplementary Methods**

*Animals*

All experiments involving mice were performed in accordance with institutional and national guidelines and regulations. *Apc*1638N animals [10] were bred with the transgenic model pVillin-*KRAS*V12G [13] in the C57Bl/6J inbred genetic background to generate compound *Apc*^1638N/+^/*KRAS*^V12G^ mice and their control littermates. Mice were maintained under a 12-hour light-dark cycle and fed with standard diet and water *ad libitum*. Genotyping was performed on DNA isolate from mouse tails as previously described [11, 13].

*Cytospin analysis of sorted cells*

After sorting by FACS, single cell suspensions were applied to glass slides by cytospin centrifugation at 500 rpm for 5 min. Cells were subsequently fixed in 2% PFA for 20 min at room temperature, washed 3 times, permeabilized by 0.2% TritonX, and blocked in 5% milk solution. Immunostaining was performed overnight at 4^0^C. A rabbit monoclonal antibody (#1247-1, Epitomics, 1:2000) was employed for intracellular staining of β-catenin.

*Immunohistochemistry (IHC) analysis*

Tissues were fixed in 4% paraformaldehyde (PFA) and embedded in paraffin. Five μm sections were mounted on slides and stained by hematoxylin and eosin (HE) for routine histology. Antibodies employed for IHC analysis include: Rabbit Monoclonal β-catenin (#1247-1, Epitomics, 1:2000), Synaptophysin (#A0010, DAKO, 1:750) and Lysozyme (A0099, DAKO, 1:15000). The Rabbit ChemMate^TM^ EnVision^TM^ kit (DakoCytomation) was employed as a secondary antibody, according to manufacturer’s instructions.

*Protein extraction and western analysis*

From each sorted population, samples of 104 cells were collected in medium (RPMI, supplemented with EGF 5 ng ml^-1^, Hydrocortisone 50 ng ml^-1^, Insulin/Transferrin supplement 1x (# 51300-044, Gibco) and 10% foetal calf serum). Cells were then washed by centrifugation at 0.8 g for 5 min at 4^0^C, and lysed in 1x NuPage LDS sample buffer (#NP0004, Invitrogen) with 50 mM dithiothreitol (DTT). Protein samples were electrophoresed using a 0.75 mm, 10% SDS polyacramide gel and transferred to an Immobilon-P transfer membrane (#IPVH00010, Millipore) by standard procedure. Membranes were blocked with odyssey blocking buffer (LI-COR, #927-40000, diluted 1:1 in PBS), incubated overnight at 4^0^C with 2 mouse β-catenin antibodies (Pan-β-catenin: #1247-1, Epitomics, 1:2000; de-phospho-specific β-catenin: ABC, clone 8E7, #05-665, Millipore, 1:500) in blocking buffer (LI-COR, #927-40000, diluted 1:1 in PBS), and labelled with fluorescent secondary antibodies Goat Anti-Mouse IgG - IRDye 680 & Goat Anti-Rabbit IgG - IRDye 800CW in blocking buffer for two color detection (LI-COR, #927-40000, diluted 1:1 in PBS). Proteins were visualized with the Odyssey scanner and analyzed with Odyssey software by using β-actin to normalize band intensities.

*Transplantation assays*

Tumour cells were sorted in medium ((RPMI supplemented with EGF 5 ng ml^-1^, Hydrocortisone 50 ng ml^-1^, Insulin/Transferrin 1x (# 51300-044, Gibco) and 10% foetal calf serum) and centrifuged at 0.8g for 5 min at 4^0^C. Cell pellets were then resuspended in a 1:1 mixture of RPMI and BD Matrigel (#356234, BD Biosciences) in a total volume of 150 μl followed by subcutaneous injection into the cranial & caudal flanks under general anaesthesia (isoflurane). Mice were monitored daily for tumour growths.

*Quantitative real-time PCR*

Intestinal tumours from 2 individual *Apc*^1638N/+^ and 3 individual *Apc*^1638N/+^/*KRAS*^V12G^ animals were pooled, digested to single cell suspensions and FACSorted for isolation of 10,000 cells from each of the Lin^-^, Lin^-^CD24^hi^CD29^+^ and Lin^-^CD24^med^CD29^+^/CD24^lo^CD29^+^ (joined gate) populations. Total RNA was isolated as described above, converted into cDNAs using High Capacity RNA-to-cDNA kit (Applied Biosystems), and employed in a pre-amplification step using the Taqman®PreAmp Master Mix (Applied Biosystems) according to manufacturer’s instructions. A total of 35 Taqman® assays for the genes listed in Supplementary Table 2 were selected from the Applied Biosystems inventoried assays. The linearity of pre-amplification was controlled for all primers. Assays were carried out as duplicate reactions using the 7900HT Fast Real Time system (Applied Biosystems). The β-actin gene (*Actb*) and glyceraldehyde 3-phosphate dehydrogenase (*Gapdh*) house-keeping genes were employed as references.

*Bioinformatic Analysis*

The arrays were scanned and cel files were uploaded into and normalised using Partek Genomics Suite 6.5 (Partek Inc., St. Louis, MO, USA, www.partek.com). The distribution of the intensity values on the individual arrays was visualized in a signal histogram and no obvious outliers were detected. The average fluorescence intensity of all annotated mouse genes was calculated using the Robust Multiarray Analysis (RMA) algorithm, including a quantile normalisation (all arrays are considered to have an equal intensity distribution) and probe sets were summarized using a median polish. Analysis of the sources of variation revealed that the fractions (Lin^-^, Lin^-^CD24^hi^CD29^+^ and Lin^-^CD24^+^CD29+/CD24^lo^CD29^+^) were the main source of variation.

To identify differentially expressed genes among the experimental groups, a three-way analysis of variance (ANOVA) was performed for each sample (Lin^-^, Lin^-^CD24^hi^CD29^+^ [P3], and Lin^-^CD24^+^CD29^+^/CD24^lo^CD29^+^ [P1+P2]) including P3 vs. Lin-, P3 vs. P1+P2, also according to the genotype of the mice they are derived from (*Apc*^1638N/+^ vs. *Apc*^1638N/+^/*KRAS*^V12G^). For each comparison between two experimental groups, the fold change of every annotated gene, together with their corresponding p-value, was exported.

Gene lists of interest (all >2-fold up-/down-regulated genes (pValue with FDR < 0.05)) were analyzed using the Ingenuity Systems Pathway Analysis Tools (Ingenuity, http://www.ingenuity.com) and Biobase Explain (Biobase, http://www.biobase-international.com) with main focus on the enrichment in GO-terms of functional processes and SwissProt keywords.

All the gene expression profiling data are available under GEO number **GSE47772** and can be viewed at: <http://www.ncbi.nlm.nih.gov/geo/query/acc.cgi?acc=GSE47772>.
